# High-Throughput Analysis of Protein Adsorption to a Large Library of Polymers Using Liquid Extraction Surface Analysis–Tandem Mass Spectrometry (LESA-MS/MS)

**DOI:** 10.1021/acs.analchem.5c01636

**Published:** 2025-06-10

**Authors:** Joris Meurs, Aishah Nasir, Grazziela P. Figueredo, Laurence Burroughs, Salah A. Abdelrazig, Chris Denning, David A. Winkler, David A. Barrett, Dong-Hyun Kim, Morgan R. Alexander

**Affiliations:** † School of Pharmacy, 6123 University of Nottingham, Nottingham, NG7 2RD, U.K.; ‡ Division of Cancer & Stem Cells, Biodiscovery Institute, 6123 University of Nottingham, Nottingham NG7 2RD, U.K.; § School of Computer Science, University of Nottingham, Nottingham NG8 1BB, U.K.; ∥ Monash Institute of Pharmaceutical Sciences, Monash University, Parkville, Victoria 3052, Australia; ⊥ Biochemistry and Chemistry, La Trobe University, Bundoora, Victoria 3042, Australia

## Abstract

Biomaterials play an important role in medicine from contact lenses to joint replacements. High-throughput screening coupled with machine learning has identified synthetic polymers that prevent bacterial biofilm formation, prevent fungal cell attachment, control immune cell attachment and phenotype, or direct stem cell fate. In-vitro preadsorption of proteins from culture medium plays a pivotal role in controlling cell response. However, there is a paucity of studies on the screening of protein adsorption into material libraries. Here, we show how quantitative analysis of protein adsorption on a 208-member polymer microarray can be achieved using liquid extraction surface analysis, combined with an adaptation of the droplet microarray (DMA) approach and tandem mass spectrometry (LESA-MS/MS) for protein identification. This study uses a fully defined cell culture medium containing only four proteins (Essential 8) to demonstrate the feasibility of the analysis approach. Our findings show that we can generate quantitative and predictive machine learning models of protein adsorption that elucidate key polymer features that describe the relationship between surface chemistry and protein adsorption. This information is of use for the rational design of new materials with bespoke protein attachment properties for biomaterials, medical devices, or in vitro compound screening.

## Graphical abstract

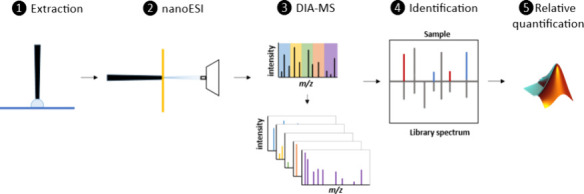

## Introduction

Biomaterials are nondrug substances derived from natural or synthetic sources that can treat, augment, or repair the body when compromised by disease or injury.^1–3^ They play an important role in medicine, e.g., contact and interocular lenses, coronary stents, joint replacements, and catheters are all widely applied. However, these have challenges, such as infections and antibiotic resistance, associated with medical devices in healthcare. Opportunities for biomaterials include the development of stem cell factories for *in vitro* toxicology models and the broad field of regenerative medicine.^1,4,5^ Given the lack of adequate theory to guide *ab initio* biomaterials development, high-throughput screening using polymer microarrays and other formats has been used extensively to identify novel biomaterials to meet these challenges and enable these opportunities.^6–13^

Current understanding of the relationships between cell attachment, cell phenotype, and materials chemistry and microtopography^8,14^ of synthetic polymeric biomaterials is incomplete, and insufficient to provide *ab initio* prediction of biomaterial performance. However, some individual systems are now well understood, e.g., polyethylene glycol protein resistance, and surfaces functionalized with peptide sequences such as RGD.^15,16^ Adsorption of culture medium-derived proteins on synthetic polymers has been shown to modulate attachment and phenotype of stem cells, blood cell adsorption and activation, and foreign body encapsulation.^17–20^ This reflects the important role of proteins from the culture medium in the response of cells to synthetic polymers and other biomaterials.

Thus far, identification of media adsorbed surface proteins on polymer microarray libraries has not been reported. However, a combinatorial experiment in which five extracellular matrix proteins were printed in array spots on a hydrogel surface to investigate their effect on stem cell attachment has been used to investigate the role of matrix protein preadsorption on cell response.^21^ Fragment fingerprints of proteins on polymer spots have been identified by secondary ion mass spectrometry (SIMS),^13^ but identification and quantification of individual proteins was not achieved due to limitations in assigning proteins using SIMS, thereby hampering the development of an understanding of the relationship between protein adsorption and physicochemical properties across a large range of diverse polymers.^22^ Improved knowledge of protein-material interactions will accelerate understanding of the mechanisms responsible for cell responses to synthetic polymer substrates, which lags behind that for more-specific protein–ligand biological interactions in ligand-functionalized materials surfaces.

Liquid extraction surface analysis-tandem MS (LESA-MS/MS) is a powerful analytical technique for the analysis of proteins using on-surface digestion.^23–27^ Combining LESA-MS with the droplet microarray (DMA) high-throughput sample format^28^ has allowed us to increase the reproducibility of the liquid surface sampling by controlling the spread of the analytical sessile drop.^29^ Therefore, the combination of LESA-MS/MS and DMA has great potential for probing the mechanisms modulating cell–surface and protein–surface interactions.

To investigate large regions of chemical space available from synthetic polymers, polymer microarrays have been particularly valuable.^7,30^ The combination of polymer microarrays and LESA as the analytical approach does not appear ideal at first, due to LESA’s inherently low spatial resolution (>0.5 mm).^31^ However, by printing superhydrophobic areas as in the DMA approach, we can overcome this limitation by trapping the polymer and the extraction solvent in a small defined area (diameter = 1.4 mm). Here, we report this as a proof-of-concept study in which polymers were chosen from previous investigations of synthetic growth substrates for human pluripotent stem cells (hPSCs).^20^ The Essential 8 (E8) medium is designed for defined stem cell culture, with incubation time chosen based on prior reports by Nasir et al.^20^ and Hammad et al.^18^ E8 is a commercially available, xeno-free, and chemically defined medium used routinely for hPSCs culture. It is a minimal-medium formulation composed of four key proteins intended to maintain pluripotency, which is necessary for hPSC attachment and survival. These are transforming growth factor beta 1 (TGFβ1), basic fibroblast growth factor (FGF2), insulin, and transferrin. The other four components of E8 medium are sodium selenate, sodium bicarbonate, l-ascorbic acid, and Dulbecco’s Modified Eagle Medium/Nutrient Mixture F-12 (DMEM/F12).^32^

While previous studies suggested that LESA-MS/MS could provide quantitative assessment of surface-adsorbed proteins, the chemical space explored was too narrow for structure–adsorption modeling, as only a 6-well plate format was used.^20^ Clearly, it is necessary to screen a much larger number of diverse polymers for protein adsorption to generate robust and predictive machine learning models that can guide design of new materials with desired biological responses.^5^ Here, we used the DMA platform combined with LESA-MS/MS analysis to screen a 208-member polymer library. We used this library to understand why different proteins adsorb to a diverse range of homopolymers and to investigate relationships between polymer surface chemistry and protein adsorption using machine learning models. Combining the materials microarray platform with high throughput mass spectrometry analysis of the deposited protein layer and machine learning provides a new avenue for biointerfacial science to study the interplay between surface chemistry and protein adsorption from complex mixtures.

## Experimental Section

### Optimization of On-Surface Digestion and LESA-MS/MS Analysis

Protein standards for bovine serum albumin (BSA; ≥97%), insulin (human recombinant insulin, ≥98%), and transferrin (*holo*-transferrin, ≥97%) were obtained from Sigma–Aldrich (Gillingham, U.K.). FGF2 (human recombinant FGF2, ≥95%) and TGFβ1 (human recombinant TGFβ1, ≥97%) were purchased from R&D Systems (Abingdon, U.K.). Sequencing grade trypsin, MS-grade trypsin (Trypsin Gold), Rapid Trypsin, and Rapid Trypsin/LysC were acquired from Promega (Southampton, U.K.). Protease stock solutions were prepared at 0.5 μg/μL in 50 mM CH_3_COOH (Optima LC-MS grade, Fisher Scientific, Loughborough, U.K.) and stored at −80 °C. Working solutions were prepared at 0.05 μg/μL in 100 mM NH_4_HCO_3_. Digestion was carried out under various conditions (time, temperatures, buffers) and described in more detail in the Results section.

### Array Preparation

Monomer solutions (*N* = 208; *n* = 3; Section S1) were prepared as 50% (v/v) or (w/v) in DMSO (≥99.5%, Honeywell, U.K.), DMSO/CHCl_3_ (HPLC grade, Fisher Scientific, Loughborough, U.K.) 1:1 (v/v) or DMSO/H_2_O (18.2 MΩ; ElgaPure, ELGA LabWater, U.K.) 1:1 (v/v) dependent on solubility. Printing solutions consisted of monomer solutions mixed with 3% (w/v) 2,2-dimethoxy-2-phenylacetophenone (99%; Sigma–Aldrich, Gillingham, UK) in IPA (HPLC grade, Fisher Scientific, Loughborough, U.K.) and DMF (99.8%, Fisher Scientific, Loughborough, U.K.) (6:4 v/v) in a 2:1 (v/v) ratio. The monomer solution was dispensed on circular superhydrophilic spots (1.4 mm diameter) of a droplet microarray slide supplied by Aquarray (Karlsruhe, Germany) using a Biodot XYZ3200 system equipped with 946PM6B pins (Arrayit, Sunnyvale, CA, USA). Spot printing was carried out under argon-rich conditions (O_2_ < 2000 ppm) and controlled at 50% relative humidity. A long-wave UV source was used to irradiate the printed spot for 30 s. Once the printing sequence had finished, all spots were irradiated for another 10 min with long-wave UV light. Then, the array was placed in a vacuum oven (Vacutherm, Thermo, U.K.) and dried for 7 days at 35 °C and <0.01 mbar. Quality checks for the arrays were performed using time-of-flight secondary ion mass spectrometry (ToF-SIMS; TOFSIMS IV, IONTOF GmbH, Münster, Germany) equipped with a 25 keV Bi^3+^ gas cluster ion beam (GCIB). The instrument was operated in raster mode. The analysis of the total was divided into three areas of 22000 μm × 18400 μm.

### Array Incubation

Essential 8 (E8) 50× supplement (Thermo Fisher Scientific, Hemel Hempstead, U.K.) was 50-fold diluted in DMEM/F-12 (Thermo Fisher Scientific, Hemel Hempstead, U.K.). The supplement contains for human (recombinant) proteins: insulin, transferrin, FGF2, and TGFβ1. The polymer array was placed in a 50 mL Falcon tube filled with Essential 8 1× and incubated for 1 h at 37 °C. After, the array was washed three times by placing it for 10 s in fresh H_2_O to remove nonadsorbing components. Subsequently, the array was dried under ambient conditions prior to protein digestion.

### On-surface Protein Digestion of Homopolymer Arrays

For protein digestion, a stock solution of 0.5 μg/μL sequencing grade trypsin (Promega, Southampton, U.K.) was prepared in 50 mM CH_3_COOH and stored at −80 °C until further use. The working solution was prepared by diluting the trypsin stock solution 10-fold with DMSO/100 mM NH_4_HCO_3_ 1:9 (v/v). The digestion solution was then distributed on the droplet microarray using the rolling droplet technique, which leaves behind sessile drops of digest solution on the polymer spots. The array was subsequently placed in a humidified chamber and incubated for 4 h at 37 °C. The humidified chamber was created in a Petri dish by placing wet tissue alongside the array and sealing the lid with wet tissue fixed on double-sided adhesive tape (Figure 1). After incubation, the array was placed in a vacuum oven (Thermo Scientific; <50 mTorr) to extract the digestion solution. The array was subsequently stored at −20 °C until further analysis.

**Figure 1:**
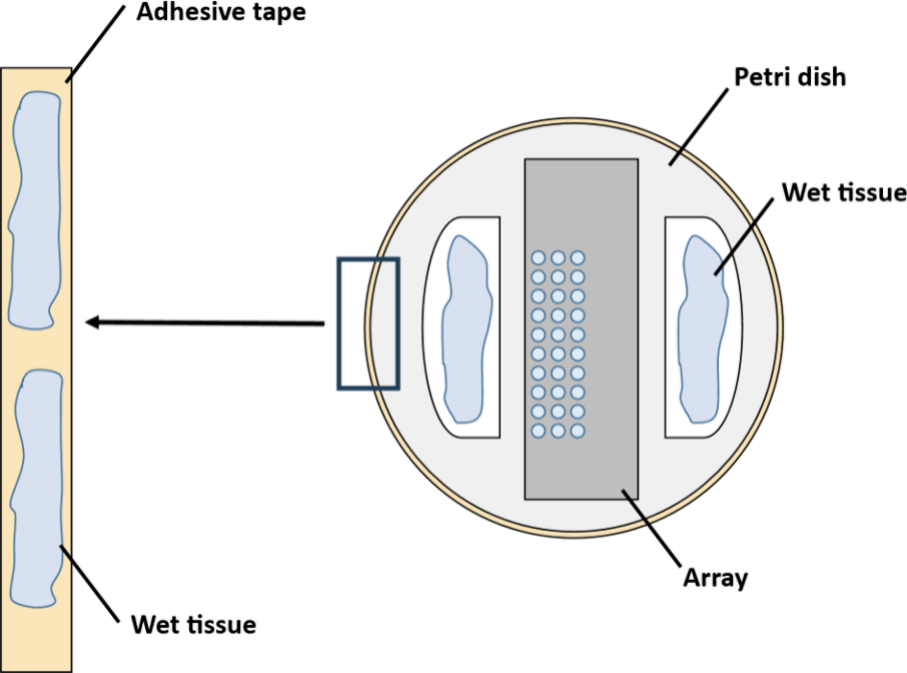
Schematic representation of the humidity chamber used for on-surface digestion. The adhesive tape containing wet tissue is placed around the rim of the Petri dish.

### LESA-MS/MS Analysis

Peptides were automatically extracted using a TriVersa Nanomate (Advion Biosciences, Ithaca, NY, USA). The extraction solvent consisted of ACN, H_2_O, DMSO, and HCOOH in respective ratios of 50:50:1:0.1 (v/v/v/v). A total of 3 μL of extraction solvent was dispensed onto the digest. After 15 s, 2 μL was aspirated back into the tip and infused into a Q Exactive plus (Thermo Scientific, San Jose, CA, USA) mass spectrometer through chip-based nanoelectrospray ionization (ESIChip, Advion Biosciences, Ithaca, NY, USA). Ionization was performed in positive mode (1.6 kV) with a 0.4 psi N_2_ back pressure.

The MS was operated in data-independent acquisition mode (DIA). For each DIA scan, a previously identified peptide ion of one of the target proteins was isolated in a 1 *m*/*z* window. Ions were fragmented using a normalized collision energy (NCE) of 27. MS^2^ data were acquired at a resolution of 35 000 (at *m*/*z* 200) with an AGC target of 1 × 10^6^ and a maximum ion injection time of 110 ms. The first mass was fixed at *m*/*z* = 100. After every 10 DIA scans, a MS^1^ scan was acquired in the range *m*/*z* 400–900 with a resolution of 140 000 (at *m*/*z* 200). For MS^1^ scans, the AGC target was set to 3 × 10^6^ with a maximum ion injection time of 200 ms.

### Protein Identification and Relative Quantification

Identification of peptide ions was done using a custom-made MATLAB (The Mathworks, Inc., Natick, MA, USA) script (https://github.com/jorismeurs/LESA_Proteomics). Briefly, Thermo.RAW files were converted to .MGF file using ProteoWizard^33^ (v3.0.1908). Subsequently, .MGF files were run through the command line versions of SearchGUI^34^ (v3.3.20) and PeptideShaker^35^ (v1.16.45) where X!Tandem,^36^ OMSSA,^37^ and MS-GF+^38^ were used as search algorithms to identify peptide ions to build a spectral library. Spectra were searched against the human proteome with 10 ppm of precursor tolerance and 0.02 Da fragment tolerance. Only fully tryptic peptides with a maximum of two miscleavages were considered. The false discovery rate was set to 1%. This spectral library was then used to identify peptides based on cosine correlation^39^ (cos θ > 0.95) and a minimum of five fragment ions. Log-transformed peptide intensities (label-free quantification) were used as a measure of protein adsorption^40^ and used for relative comparison of protein adsorption across homopolymers. A schematic of the workflow is shown in Figure 2.

**Figure 2:**
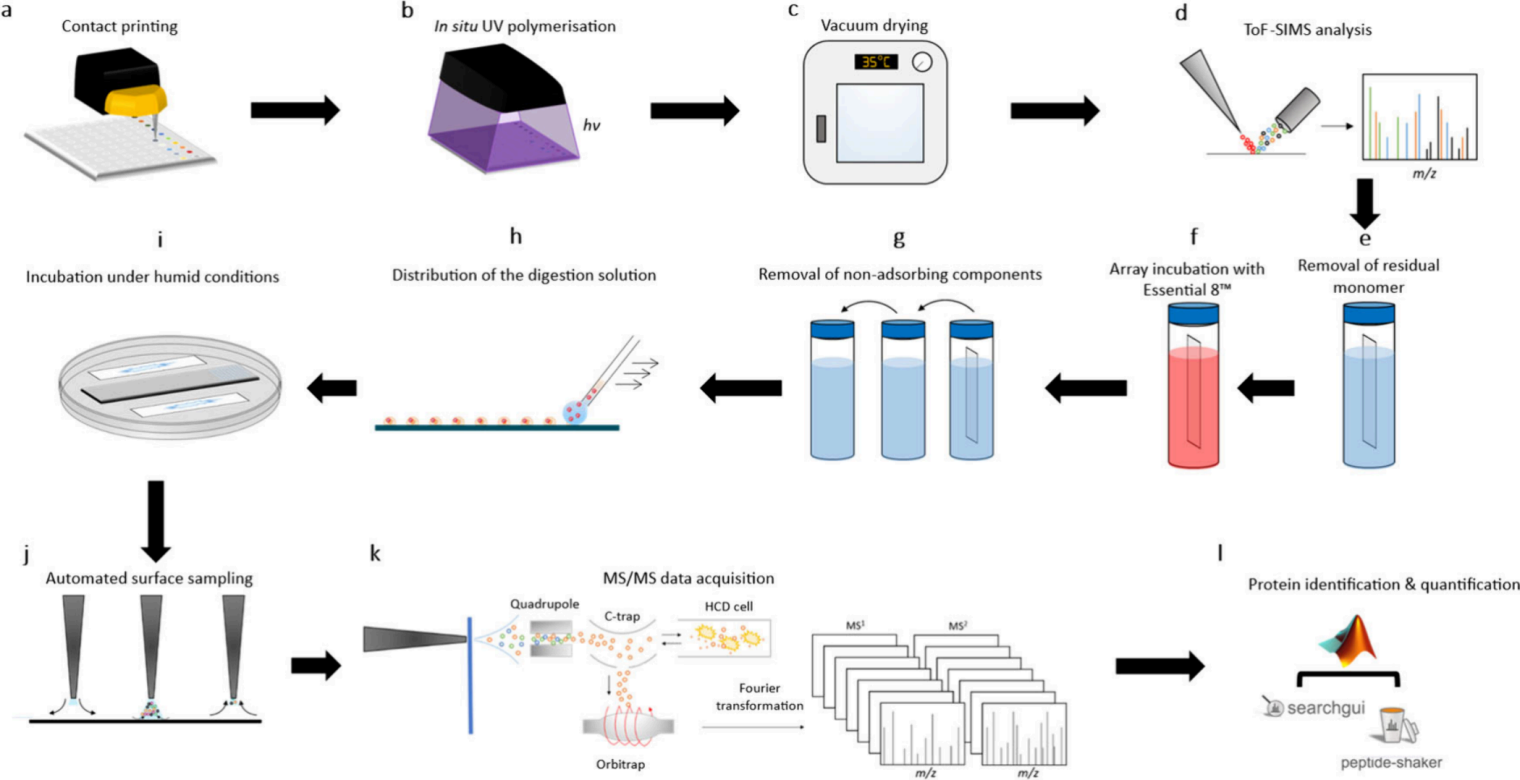
Schematic representation of the workflow for the microarray production and subsequent analysis of protein adsorption using LESA-MS/MS. (a) Through contact printing and (b) subsequent in-situ polymerization and polymer microarray is created. (c) This is array is thereafter dried for 7 days in a vacuum oven at 35 °C. (d) ToF-SIMS analysis is performed to confirm the presence of the polymers. (e) After, residual monomer is removed by rinsing the array with deionized water. (f) This was followed by incubation of the array in Essential 8 medium. (g) Nonadsorbing compounds were removed after which (h, i) on-surface digestion was performed. (j, k) Digests are then analyzed with LESA-MS/MS and (l) identified and quantified using SearchGUI, PeptideShaker, and MATLAB.

### Modeling Protein–Chemistry Relations

Dragon descriptors^41^ and fragment descriptors^42^ encoding relevant molecular properties of the polymers were used to train machine learning models of protein adsorption. Missing peptide intensities were replaced with the threshold value for peak intensity, which was set to 1000. To deal with large differences between replicates, geometric means were used and only adsorption values with a signal-to-noise ratio (SNR) of >1.5 were included for modeling. LASSO feature selection was performed to remove low relevance features to avoid overfitting and to aid interpretation. Sparse multiple linear regression models with expectation maximization models were trained on these features. Model predictivity was assessed by using a test set. Training and test dates were split into proportions of 80% and 20%, respectively, using 50 bootstraps. Prediction models for each protein were assessed using root mean squared error (RMSE) and the coefficient of determination (R^2^). The size and sign of the contribution of the sparse features were extracted from regression coefficients and were used to infer relations between polymer chemistry and protein adsorption.

## Results and Discussion

LESA-MS/MS has not previously been applied to polymer microarray analysis. Therefore, we first optimized and validated the data acquisition parameters and protocols for the four proteins in the E8 medium on adsorption to surfaces.

### Optimization of On-Surface Digestion

In a previous study, the feasibility of LESA-MS for the analysis and relative quantification of proteins adsorbed to biomaterial surfaces has been demonstrated.^20^ However, the methodology has not yet been explored for screening a large library of polymers. To ensure high-quality data, we first optimized the on-surface digestion conditions to achieve reproducible results. A previous protocol developed by Rao et al.^23^ used sequencing-grade trypsin at room temperature to achieve digestion of proteins on surfaces to peptides for ready mass-spectrometric analysis. We chose to use insulin to study the digestion efficiency using different grades of proteases and incubation temperature. This can be readily studied for insulin because only one tryptic peptide (GFFYTPK) is formed and both the peptide and the intact protein charge states 4+ and 5+ can be monitored in a single mass-to-charge ratio range (Section S2). On-surface digestion was performed with Trypsin Gold, Trypsin/LysC, and sequencing-grade trypsin at 37 °C. On-surface digestion with Rapid Trypsin and Rapid Trypsin/LysC was done for 4 h at 70 °C. It was observed that overnight on-surface digestion outperformed Rapid Trypsin on-surface digestion at elevated temperature (Rapid Trypsin vs sequencing-grade trypsin; see Figure 3a). One of the main issues with digestion at higher temperatures is the evaporation of droplets, quenching the digestion. At 37 °C, the volume of the droplets containing the protease did not seem to be significantly affected during digestion. However, droplet volumes were not measured after incubation. No mixing is expected between sessile digesting solution droplets due to their hydrophobic separation. Furthermore, adding DMSO to an end concentration of 10% (v/v) to the digestion buffer showed also to enhance the digestion efficiency based on the higher peptide signal intensity for GFFYTPK (Figure 3b; *p* < 0.001) and an increased peptide/intact protein ratio, compared to a digestion buffer without DMSO (*p* = 0.015). The increased digestion efficiency can be explained by improved solubilization of hydrophobic proteins.^43^ Moreover, DMSO has been reported as an enhancer for trypsin activity.^44^ Using other solvents or surfactants could be of interest, especially when dealing with more-complex mixtures to improve solubilization in the droplet. It should be however investigated whether these solvents and/or additives are compatible with the analysis, as residues might impact electrospray stability.^45–47^

**Figure 3:**
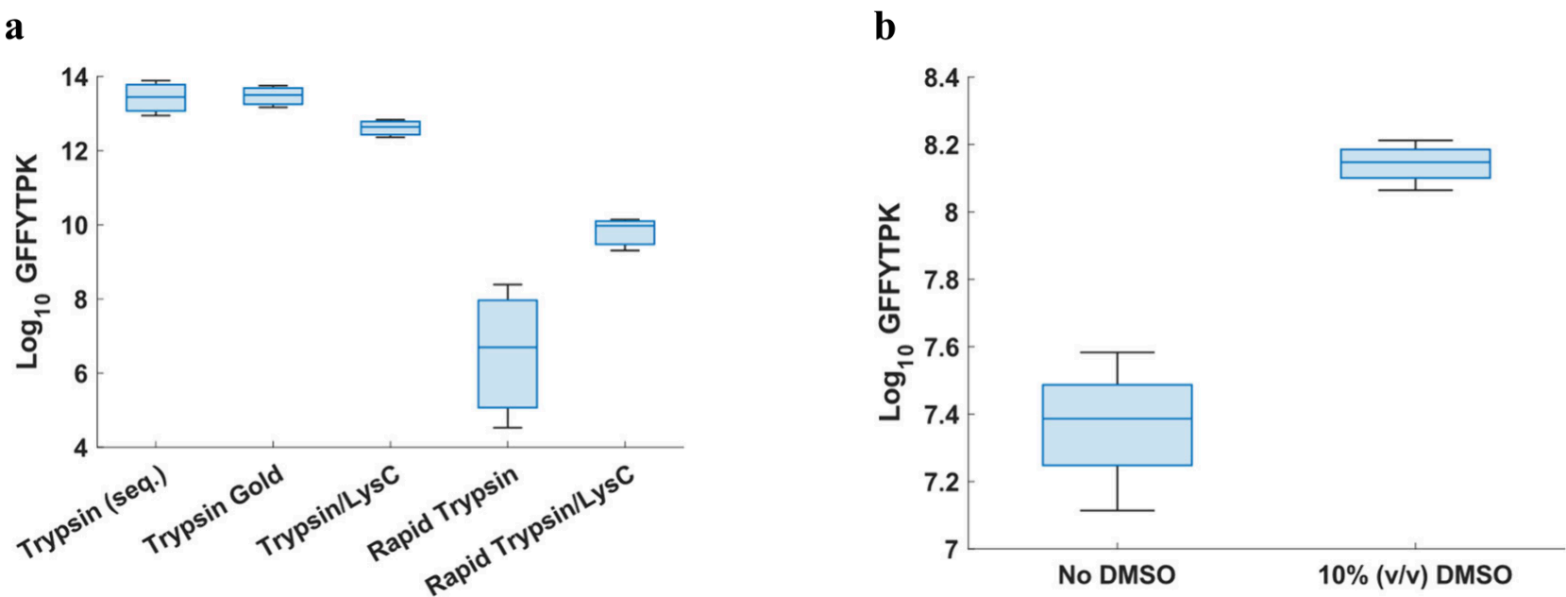
Optimization of on-surface digestion conditions. (a) Log_10_ transformed intensities for GFFYTPK after digestion with different proteases (*n* = 3). (b) Investigating the benefit of DMSO as a digestion additive (*n* = 4). Box and whiskers denote the quartiles of the data.

### Optimization of LESA-MS/MS Parameters

LESA parameters were optimized by using an in-solution digest of BSA. The investigated parameters per optimization experiment are shown in Table 1.

**Table 1:** Selected LESA Parameters for Optimization

LESA parameter	settings
extraction solvent mixtures	ACN/H_2_O (1:1 v/v); ACN/H_2_O/HCOOH (50:50:0.1 v/v/v); ACN/H_2_O/HCOOH/DMSO (50:50:0.1:1 v/v/v/v)
	
extraction volumes	1.0 μL, 1.5 μL, 2.0 μL, 2.5 μL

Rao et al.^23^ suggested ACN/H_2_O 1:1 (v/v) for extracting peptides from polymer surfaces. Formic acid has been reported as a LESA extraction solvent additive for proteomics experiments,^48^ as it is thought that weak organic acid modifiers improve the ionization of basic functional groups.^49^ The addition of formic acid, HCOOH (0.1%, v/v) in ACN/H_2_O (1:1, v/v) significantly reduced variability, as quantified by the coefficient of variance (CV) for BSA peptides, decreasing it from 118% to 49% (*p* < 0.001) between replicate on-surface digests. HCOOH is known to improve ionization of peptides,^50^ and this could explain the improved signal stability of the BSA peptides. The addition of HCOOH also had an enhancing effect on the signal intensity for BSA peptides (*p* < 0.001; Figure S3A) and improved the stability of the total ion current (*p* < 0.001). It was found that a minimum volume of 1.5 μL extraction solvent was required to achieve a stable total ion current (CV < 15%; Figure S3B). Further increasing the solvent volume did improve the signal stability; however, increasing the solvent volume beyond 1.0 μL had no statistically significant effect.

Previous reports have shown the benefit of using DMSO as an additive solvent to enhance the ionization of hydrophobic peptides.^51–53^ Here, the addition of DMSO was tested for its effect on the extraction and ionization of BSA peptides. The addition of 1% (v/v) was found in the overall peptide intensity distribution (*p* = 0.3501; Figure S3C), indicating no enhanced effect of DMSO for peptides during LESA-MS/MS analysis. However, it was found that the use of 1% (v/v) DMSO in ACN/H_2_O/HCOOH 50:50:0.1 (v/v/v) improved (*p* < 0.001) the signal repeatability, on average, from 4.0% to 1.9%, compared to solely ACN/H_2_O/HCOOH 50:50:0.1 (v/v/v) (Figure S3D). The improved repeatability might be attributed to the better solubility of the peptides. Using DMSO as an additive has been shown to reduce the relative standard deviation in a label-free quantification experiment, which is consistent with our findings.^54^

### Predicting Protein Adsorption from Molecular Features Using Machine Learning

Microarrays were produced in triplicate containing 208 unique homopolymer chemistries on a droplet microarray. Microarrays were incubated in Essential 8 cell culture medium for protein adsorption. Subsequently, in-situ digestion was performed and the optimized LESA-MS/MS method was used to assess relative protein adsorption (log_10_ transformed summed peptide intensities) across the diverse chemical range of polymers to better understand protein–polymer interactions. The protease solution was introduced on DMA using the rolling droplet technique. The exact volume of the protease solution on a single superhydrophilic spot was not been measured. Assuming a spherical shape of the droplet^55^ and a contact angle of 5.2°,^56^ the droplet volume was found to be 0.19 nL. The droplet-to-droplet volume variation is expected to be within 10%.^28^ The total liquid extraction time per polymer spot was 15 s, and data were acquired for the duration of 2 min. This resulted in a total analysis time of approximately 8 h for one array. The subsequent data processing for extracting peptide intensities from LESA-MS^1^ spectra took approximately 4.5 min.

Protein adsorption was found to vary substantially among the polymers. The protein most strongly adsorbed was insulin (Figure 4a). Insulin is also the most abundant protein in the E8 medium. Studying the monomer structures of the top 10 strongest adsorbing polymers per protein already reveals insights in chemistry-protein interactions (Figure 4b). Transferrin appears to prefer acrylamide groups while fluorinated polymers tend to promote stronger adsorption of insulin and FGF-2.

**Figure 4:**
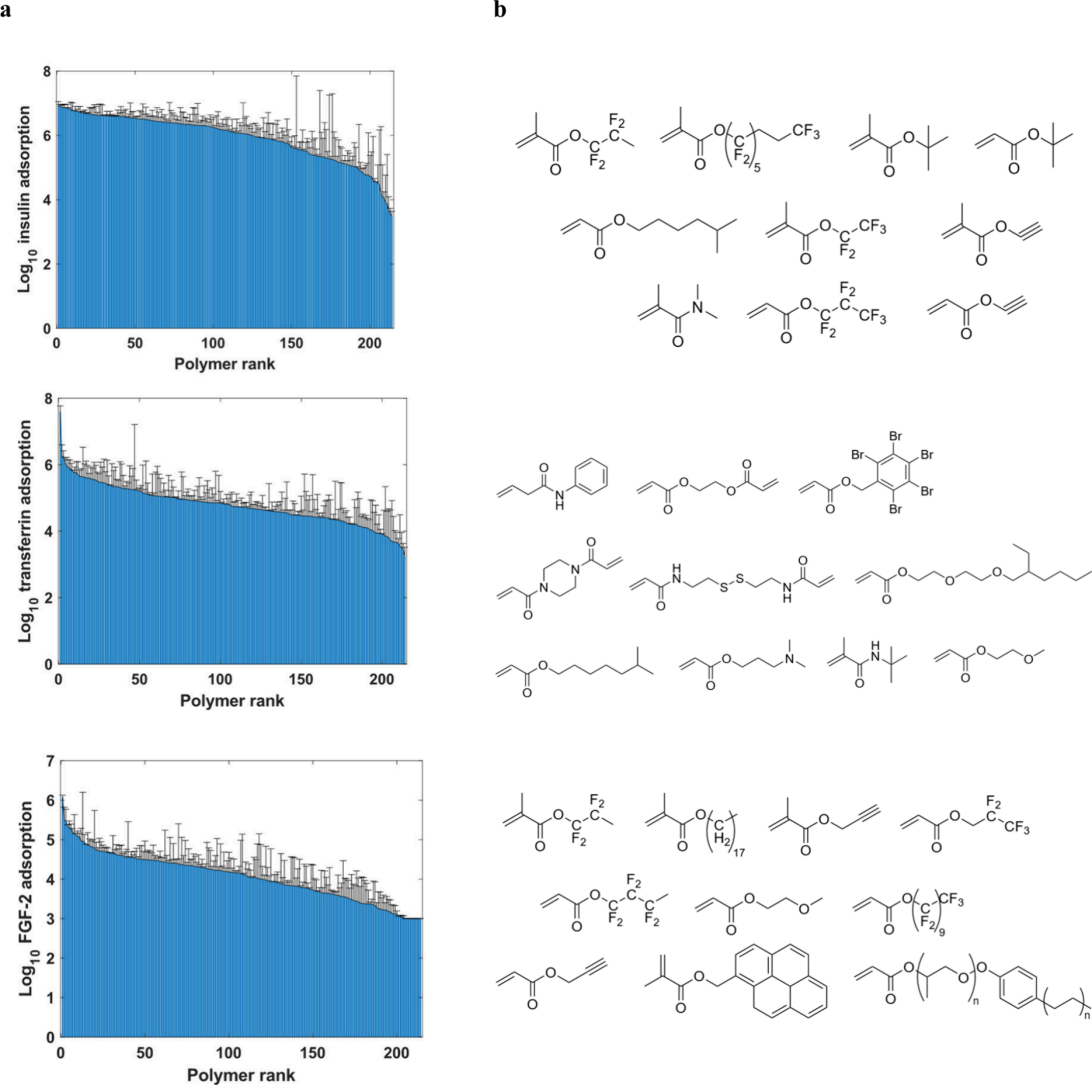
(a) Bar graph displaying ranked average protein adsorption as log_10_-transformed peptide intensity across polymers, ranked separately for insulin (top), transferrin (middle), and FGF-2 (bottom). Error bars represent one standard deviation of the mean. (b) Monomer structures for top 10 highest adsorption for insulin (top), transferrin (middle), and FGF-2 (bottom).

To understand protein–chemistry interactions, multiple linear regression models with expectation maximization were generated for insulin, transferrin, and FGF2 using Fragment and Dragon descriptors of the polymer structures.^41,42^ Prior studies have shown that instructive properties can be derived from cell–polymer interactions using machine learning.^8,9,14,57,58^ Therefore, a similar approach was used here to obtain an understanding of the protein–material interactions.

Prior to training the model, polymers which showed a large variation (SNR < 1.5) across replicates were removed from the dataset. This was done to ensure that the derived model reflects true adsorption behavior rather than measurement noise. Out of 208 polymers, 70, 45, and 60 polymers remained after SNR filtering for insulin, transferrin, and FGF-2, respectively. LASSO was used to select predictive features prior to modeling. Earlier reports have shown the feasibility of modeling with chemical descriptors to understand the interaction of bacteria and mammalian cells.^57^ The outcome of the test set and the regression coefficients for the significant descriptors are listed in Figure 5.

**Figure 5:**
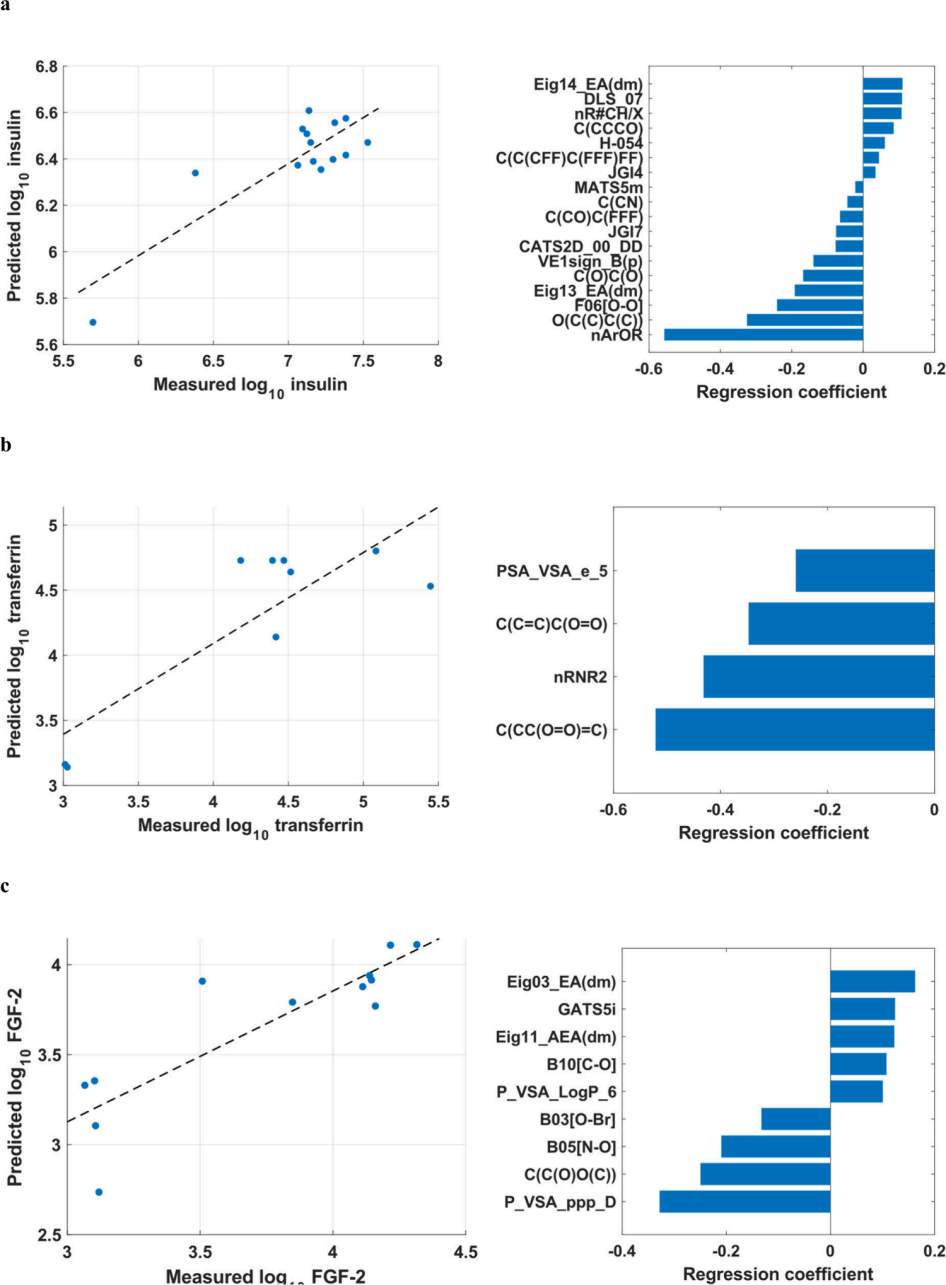
Multiple linear regression with maximum expectation modeling for the test set of (A) insulin (*R* ^2^ = 0.63), (B) transferrin (*R* ^2^ = 0.55), and (C) FGF-2 (*R* ^2^ = 0.62). Figures on the left represent the prediction error, and figures on the right represent the regression coefficients for significant molecular descriptors.

An overview of significant fragment descriptors is given for each protein in Table 2. A larger number of significant Dragon descriptors was identified and described in Table S4. The interpretation of the Dragon descriptors is not intuitive, relative to the protein–chemistry mechanisms. However, the identification of a model first indicates that there is a relationship with the chemistry of the monomers, and second, subsequent monomers for screening can be chosen rationally based on these descriptors to increase/decrease protein adsorption. Here, we further focus on the fragment descriptors in relation to protein adsorption.

**Table 2:**
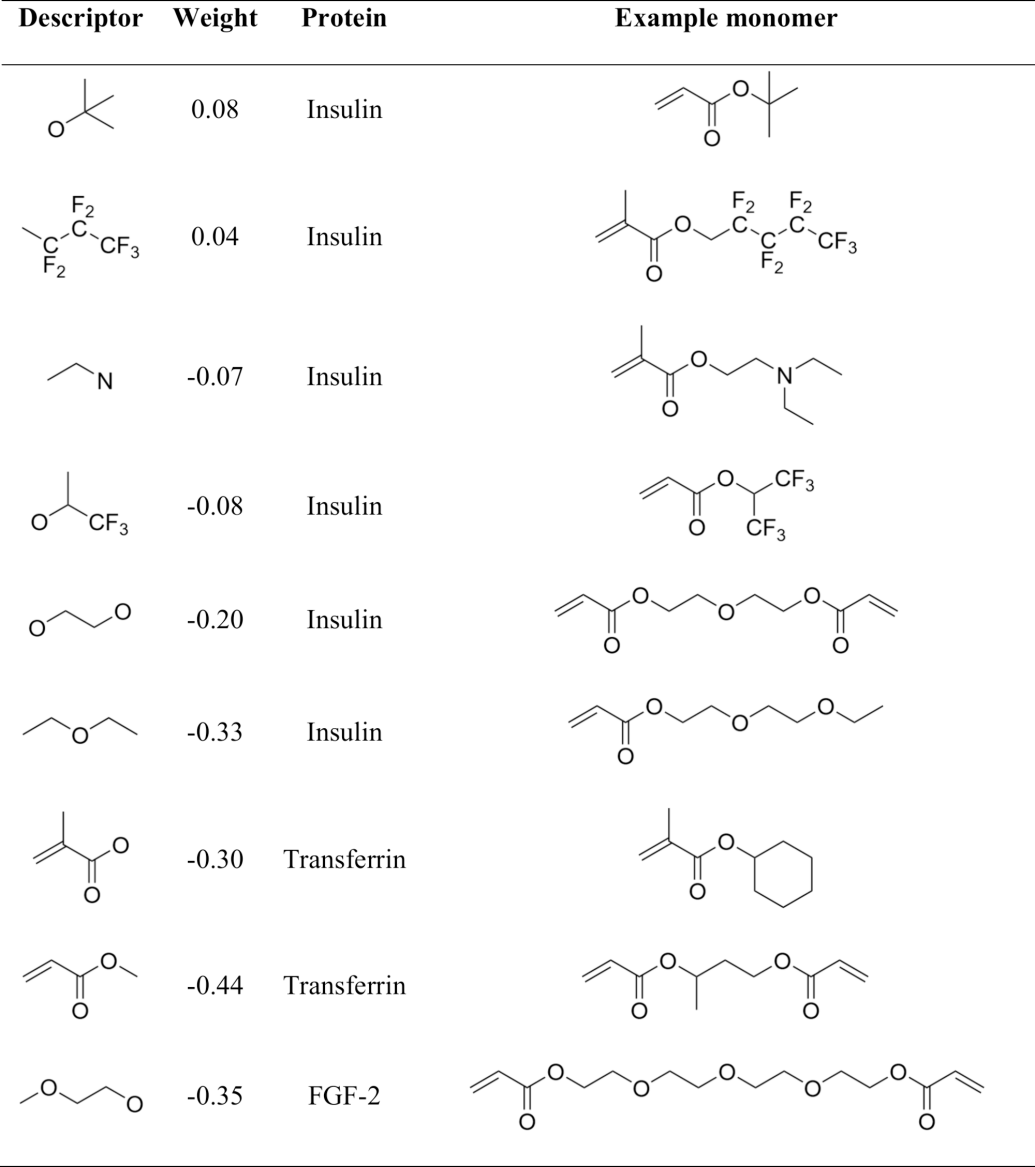
Signature Descriptors with the Greatest Impact on Protein Adsorption, Positive Regression Coefficients Increase Protein Adsorption, Negative Regression Coeffcients Decrease Protein Adsorption

The role of insulin in cell culture is to support survival and proliferation.^32^ Insulin is a small (5.8 kDa) hydrophobic protein^59^ and would therefore, through hydrophobic–hydrophobic interactions, prefer adsorption onto hydrophobic surfaces. That is found to be the case by the positive regression coefficients for the C­(CCCO) and C­(C­(CFF)­C­(FFF)­FF) fragment descriptors, which have a *tert*-butyl moiety and a fluorine-rich structure, respectively (Figure 5a). In addition, more hydrophilic entities such as ethylene glycol moieties (C­(O)­C­(O)) and ethers (O­(C­(C)­C­(C)) are shown to correlate with low adsorption (negative regression coefficient) of insulin. A study by Mollmann et al.^60^ showed the adsorption behavior of insulin on Teflon particles. It was demonstrated that insulin undergoes conformational changes upon being adsorbed to the surface. These conformational changes might be important for subsequent adsorption of (mammalian) cells.

Transferrin is added to medium to increase the survival rate and cloning efficiency of hESC and hPSCs.^32^ While the top 10 strongest transferrin-adsorbing polymers were shown to contain amine groups, this was not found to be a significant fragment descriptor by the model (Figure 5b). This can be explained by the filter step excluding large variation (SNR < 1.5) in transferrin adsorption to not bias the model. The regression model only returned negative regression coefficients, indicating its low adsorption. Moreover, the number of aliphatic tertiary amines (nRNR2) has a negative influence on transferrin adsorption. Interestingly, the fragment descriptors C­(C=C)­C­(O=O) and C­(CC­(O=O)­C) refer to methacrylate and acrylate, which is the backbone of each polymer. Transferrin is a protein of approximately 76 kDa with both hydrophobic and hydrophilic sites.^61^ Therefore, the protein orientation to the surface could play a role. Proteins that adsorb to an interface do readily undergo structural changes to favor adhesion.^60^

Similar to insulin, FGF-2 (18 kDa) plays an important role in the proliferation and differentiation, across various mammalian cells.^32,62^ For FGF-2, only one fragment descriptor was found to significantly contribute to the regression model (Figure 5c). The ethylene glycol moiety C­(C­(O)­O­(C)) reduces the amount of FGF-2 that can adsorb to the surface. No significant fragment descriptors were found that contribute to promoting FGF-2 adsorption. It could have been expected that fluorine-containing fragment descriptor would show positive regression coefficients as (per)­fluoropolymers appeared in the top 10 highest FGF-2 adsorption. Also here, this can be attributed to the variation (SNR < 1.5) between replicates which caused several (per)­fluoropolymers to be filtered out prior to training the machine learning model. Nonetheless, a log *P* Dragon descriptor (P_VSA_LogP_6) contributes positively to FGF-2 adsorption indicating a significant role for protein hydrophobicity.

The tertiary structure of a protein is highly dependent on its microenvironment, such as the polymer surface, and has a significant influence upon cell response.^19,59^ Not only protein quantity,^18^ but also conformation and/or orientation of the protein on the surface is vital for, e.g., cell adhesion.^19,63^ If proteins, like the ones studied here, undergo conformational changes to adsorb to a different chemical interface, quantifying the amount of adsorbed protein is a first step in understanding adsorption, but including protein conformation would be essential in future studies to further improve the understanding of protein–surface chemistry interactions. Also, the orientation of the protein could be influenced based on what site it adsorbs to the polymer substrate. Further developing this high-throughput surface analysis method would be required going toward strategies like hydrogen-deteurium exchange (HDX) to gain more insights into protein orientation and/or conformation on a polymer surfaces.^64^ As currently a large number of polymers displayed highly variation (SNR < 1.5) in protein adsorption, the covered chemical space is small. Having crucial information on protein orientation would also be valuable for designing a fit-for-purpose robust relative quantification method.

Understanding the quantitative adsorption of proteins on synthetic substrates is crucial for optimizing biomaterials for a range of regenerative medicine applications. This insight into proteins with a wide range of polymers in an array format is an indication that the combination of LESA-MS/MS analysis and machine learning can provide valuable insights into chemistry-driven protein adsorption, which could aid understanding protein–chemistry interactions and eventually rationalizing the design of new biomaterials. Protein adsorption governs the biochemical and biophysical properties of the substrate, directly influencing cell adhesion, proliferation, and differentiation. By quantitatively characterizing how these proteins interact with synthetic surfaces, researchers can design biomimetic environments through substrate selection, e.g., SureCoat,^65^ Corning Synthemax,^66^ or medium supplements, e.g., interalpha-inhibitor^67^ or Physiologix.^68^ Additionally, precise control over protein adsorption enables the development of scalable and reproducible culture platforms e.g. cultivated meat manufacturing critical^69^ for both fundamental research and clinical translation such as to inform biocompatibility of medical device implants^70,71^ for regenerative medicine applications such as wound healing^72^ and implant devices.^73^ In addition, it became evident that protein orientation and/or conformational changes are essential to study to gain a fundamental understanding of how protein interactions on a synthetic polymer surface influence cell response.

## Conclusions

We have successfully developed and validated a LESA-MS/MS analysis method for high-throughput quantitative analysis of adsorbed proteins on a library of synthetic polymers. In addition, with the combination of machine learning, this strategy is a useful tool for the high-throughput screening of protein–material interactions and can be extended to more-complex cell culture media and biological fluids. This provides a basis for revealing relationships between quantitative protein adsorption and cellular response in high-throughput studies to provide mechanistic biointerfacial insight.

## Supplementary Material



## Data Availability

All data are available on FigShare (URL: www.doi.org/10.6084/m9.figshare.28608410).

## Notes

Section S1: Overview of monomer structures used for fabricating polymer arrays; Section S2: LESA-MS^1^ spectrum for an insulin tryptic on-surface digest; Section S3: Optimization of digestion and LESA parameters for the analysis of peptides; Section S4: Overview of significant Dragon descriptors (PDF)

## ∇

For J.M.: Life Science Trace Detection Laboratory, Department of Analytical Chemistry & Chemometrics, Radboud University, Nijmegen 6525 AJ, The Netherlands

## ○

For S.A.A.: Marine Microbiomics, New York University Abu Dhabi, PO Box 129188, Abu Dhabi C1–013, United Arab Emirates.
