# Supplementary material for: High-Throughput Analysis of Protein Adsorption to a Large Library of Polymers Using Liquid Extraction Surface Analysis–Tandem Mass Spectrometry (LESA-MS/MS)
Source: Anal Chem. 2025 Jun 10;97(24):12776–85. doi: 10.1021/acs.analchem.5c01636 (PMC12199226; doi:10.1021/acs.analchem.5c01636)
Supplement: Supplementary file 1 [file ac5c01636_si_001.pdf]

Supporting Information

High throughput analysis of protein adsorption to a large library of polymers using liquid extraction surface analysis – tandem mass spectrometry (LESA-MS/MS)

Joris Meurs[1], Aishah Nasir[1,2], Grazziela P. Figueredo[3], Laurence Burroughs[1], Salah A. Abdelrazig[1], Chris Denning[2], David Winkler[1,4,5], David A. Barrett[1], Dong-Hyun Kim[1] & Morgan R. Alexander[1]*

[1]: *School of Pharmacy, University of Nottingham, Nottingham, NG7 2RD, United Kingdom*

[2]: *Division of Cancer & Stem Cells, Biodiscovery Institute, University of Nottingham, Nottingham NG7 2RD, UK*

[3]: *School of Computer Science, University of Nottingham, Nottingham NG8 1BB, UK*

[4]: *Monash Institute of Pharmaceutical Sciences, Monash University, Parkville, Victoria 3052,Australia*

[5]: *Biochemistry and Chemistry, La Trobe University, Bundoora, Victoria 3042, Australia*

Table of content

S1: Overview of monomer structures used for fabricating polymer arrays

S2: LESA-MS[1] spectrum for an insulin tryptic on-surface digest

S3: Optimisation of digestion and LESA parameters for the analysis of peptides

S4: Overview of significant Dragon descriptors

Supplement S1: Overview of monomer structures used for fabricating polymer arrays

Acronym	Name	Structure
13BDDA	Butanediol-1,3 diacrylate	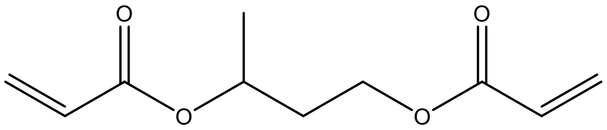
AAcAm	Diacetone acrylamide	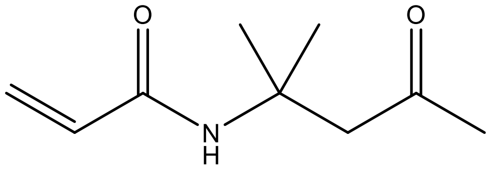
AcAPAm	N-[2-(Acryloylamino)phenyl]acrylamide	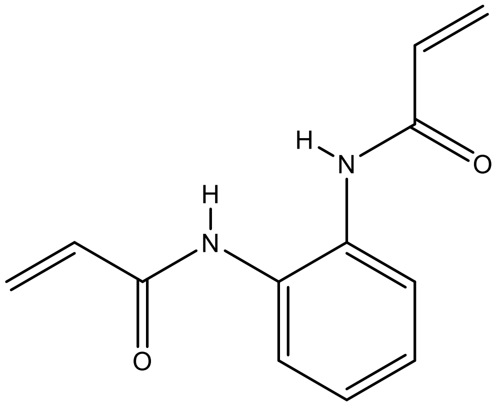
AnMA	Anthracenylmethylacrylate	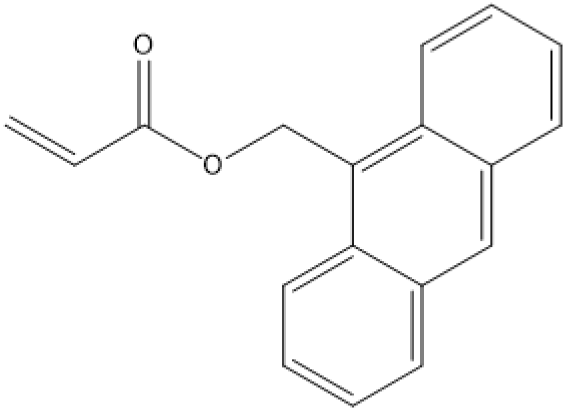
BnMA	Benzyl methacrylate	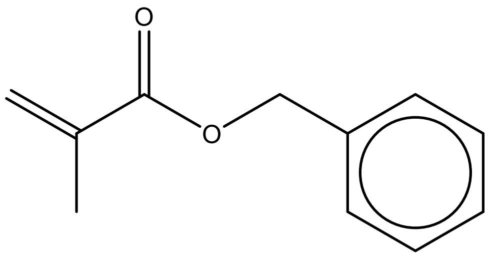

Acronym	Name	Structure
BPEODA	Bisphenol A ethoxylate diacrylate	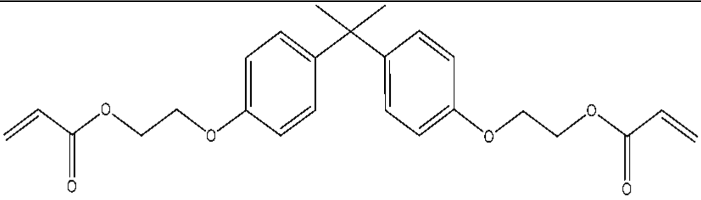
BzHPEA	Benzoyl-3-hydroxy-phenoxy)ethyl acrylate	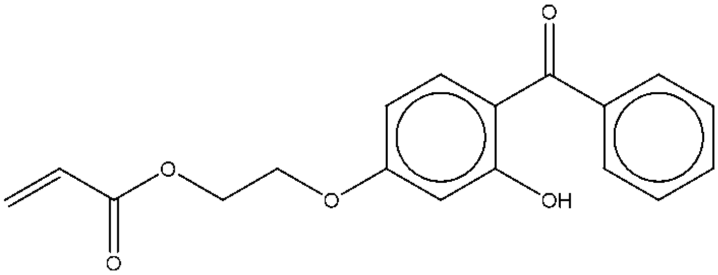
CHMA	Cyclohexyl methacrylate	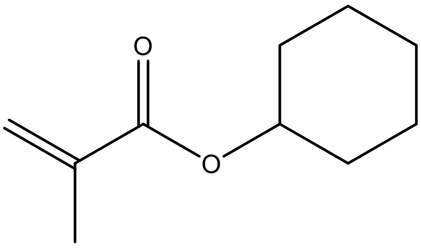
CMAOE	Caprolactone 2-(methacryloyloxy)ethyl ester	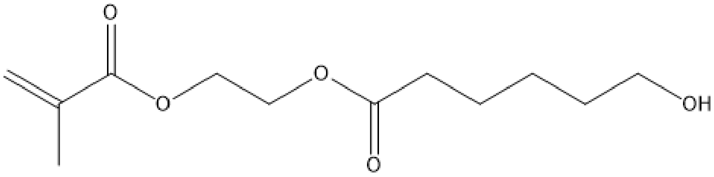
CNEA	Cyanoethyl acrylate	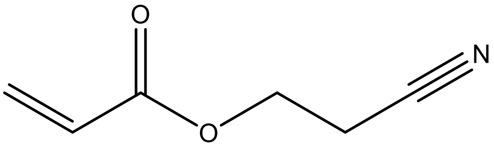
DEAEMA	Diethylaminoethyl methacrylate	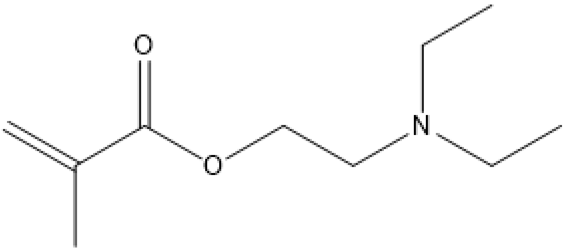

Acronym	Name	Structure
DEGDMA	Diethylene glycol dimethacrylate	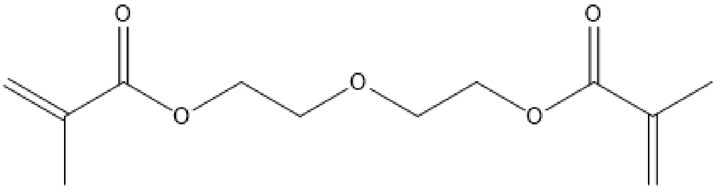
DEGEEA	Di(ethylene glycol) ethyl ether acrylate	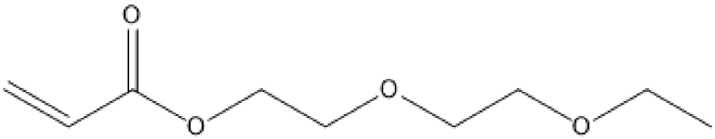
DEGEHA	Di(ethylene glycol) 2-ethylhexyl ether acrylate	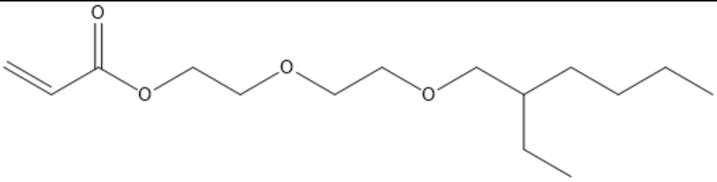
EaNIA	Ethyl trans-a-cyano-3-indole-acrylate	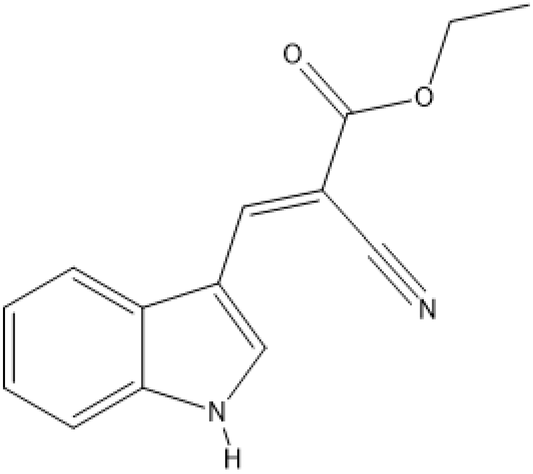
EbCNA	Ethyl-cis-B-cyano-acrylate	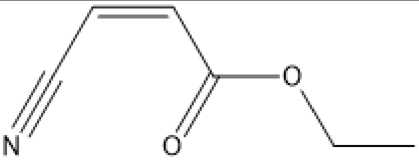
EG4DMA	Tetraethylene glycol dimethacrylate	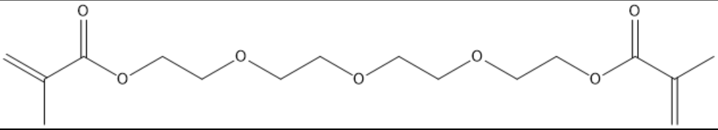
EGDPEA	Ethylene glycol dicyclopentenyl ether acrylate	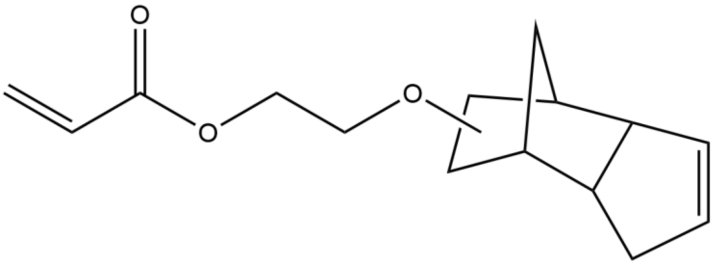

Acronym	Name	Structure
EGDMA	Ethylene glycol dimethacrylate	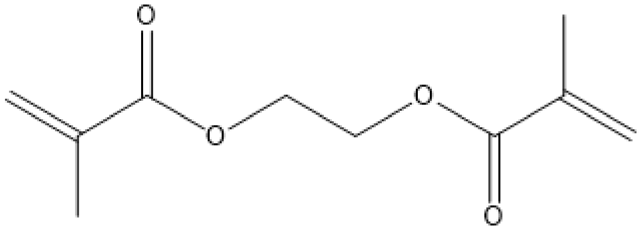
EGMEA	Ethylene glycol methyl ether acrylate	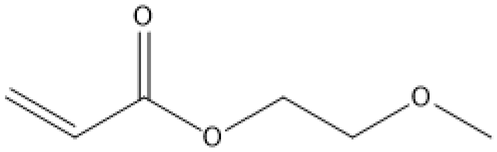
EGPEA	Ethylene glycol phenyl ether acrylate	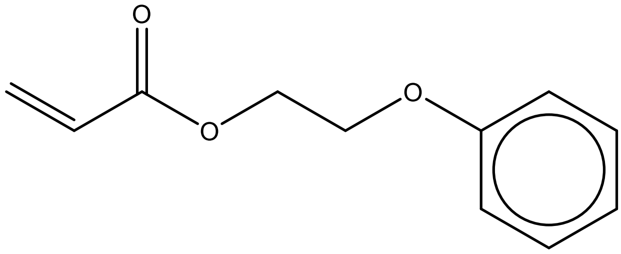
EGPhMA	Ethylene glycol phenyl ether methacrylate	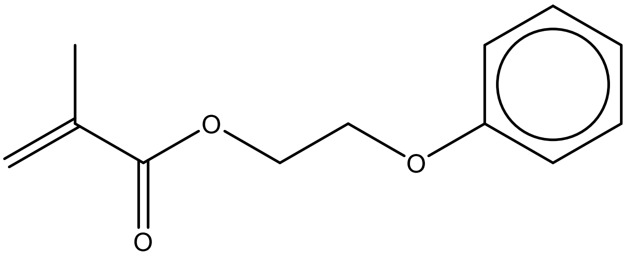
EOEA	Ethoxyethyl acrylate	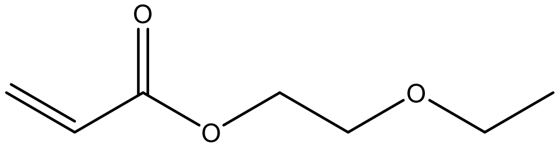
FMHPNMA	Trifluoro-2’-(trifluoromethyl)-2’-hydroxy)propyl]-3-norbornyl methacrylate	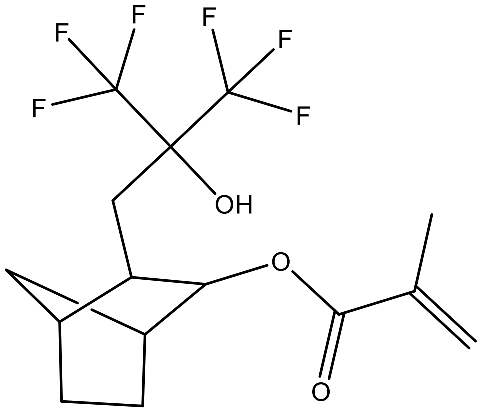

Acronym	Name	Structure
HDDMA	1,6-Hexanediol dimethacrylate,	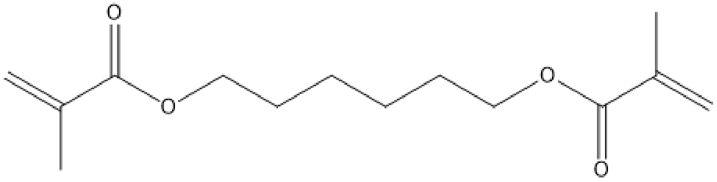
HFiPA	Hexafluoroisopropyl acrylate	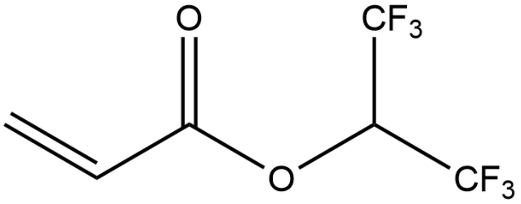
HFiPMA	Hexafluoroisopropyl methacrylate	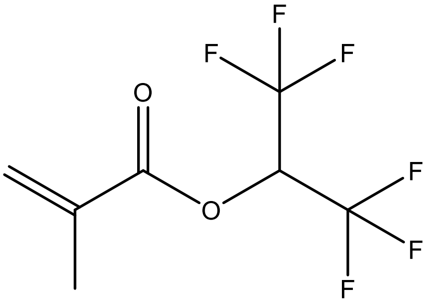
HMA	Hexyl methacrylate	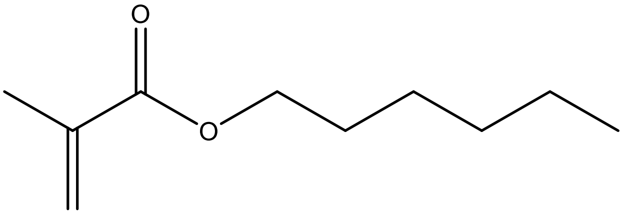
HPA	Hydroxypropyl acrylate	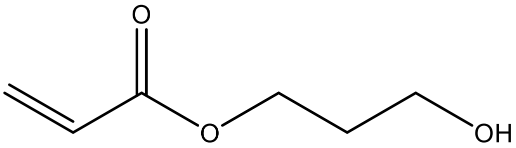
iBA	Isobutyl acrylate	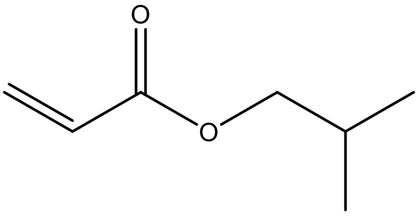
iBMA	Isobornyl methacrylate	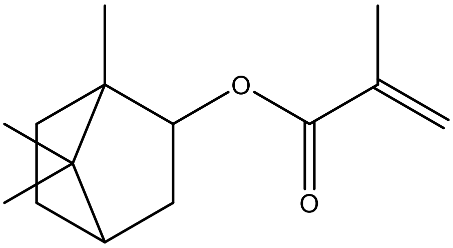

Acronym	Name	Structure
iBOA	Isobornyl acrylate	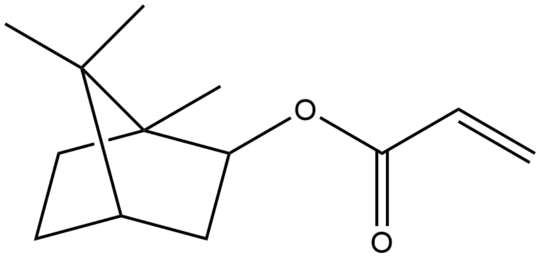
iBOMAm	N-(Isobutoxymethyl)acrylamide	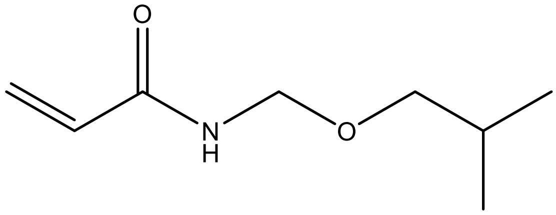
iDA	Isodecyl acrylate	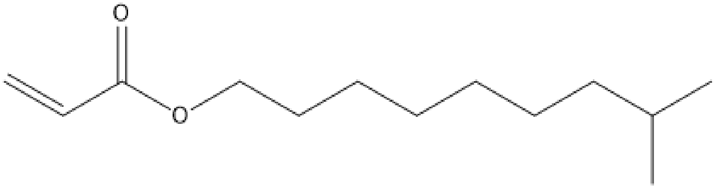
iDMA	Isodecyl methacrylate	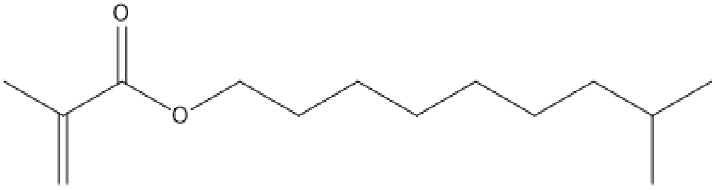
LaA	Lauryl acrylate	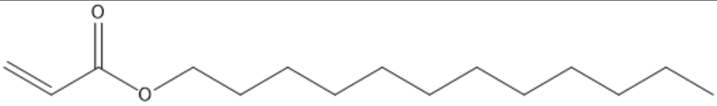
LMA	Lauryl methacrylate	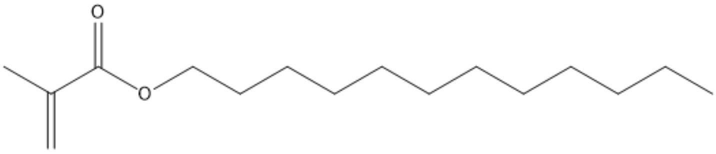
MAA	Methyl 2-acetamidoacrylate	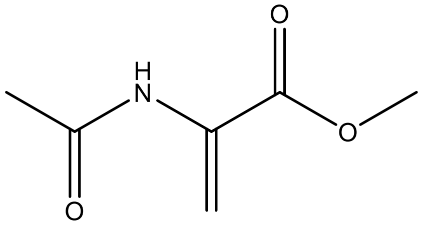

Acronym	Name	Structure
MAAH	Methacrylic anhydride	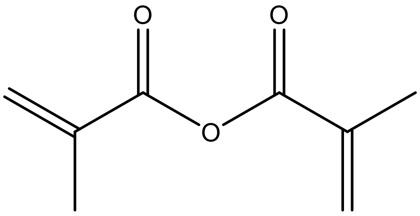
MAEA	Methacryloyloxy)ethyl acetoacetate	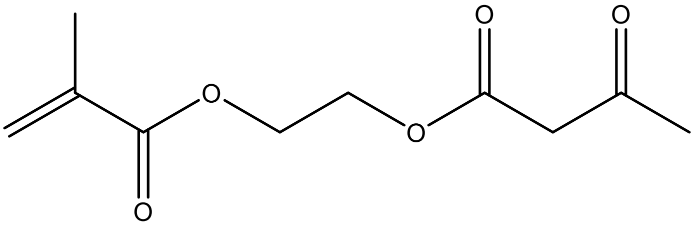
MAL	Methacryloyl-L-Lysine	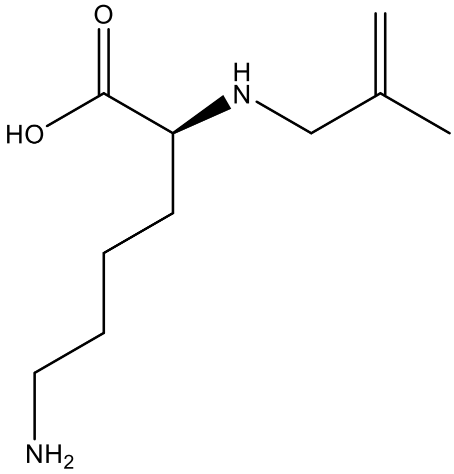
Mam	Methacrylamide	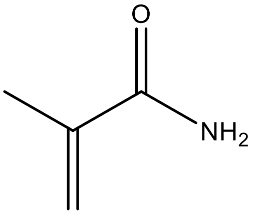
MBMAm	N,N'-Methylenebismethacrylamide	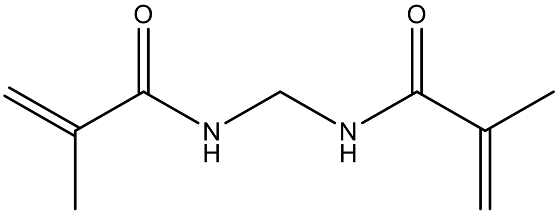

Acronym	Name	Structure
MEDMSAH	[2-(Methacryloyloxy)ethyl]dimethyl-(3-sulfopropyl) ammonium hydroxide	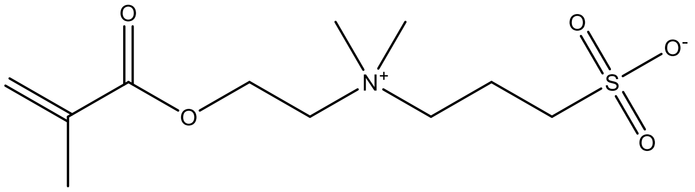
MMAm	N-Methylmethacrylamide	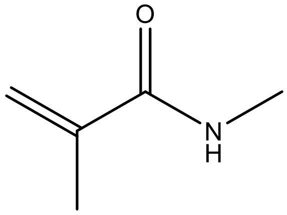
MPDSAH	Methacryloylamino)propyl]dimethyl(3-sulfopropyl)ammonium hydroxide inner salt	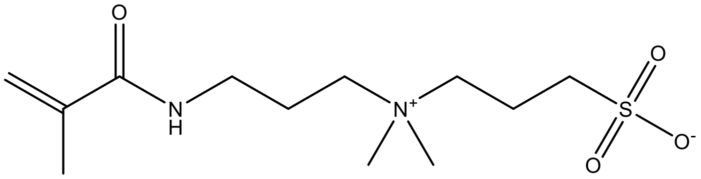
MTEMA	Methylthioethyl methacrylate	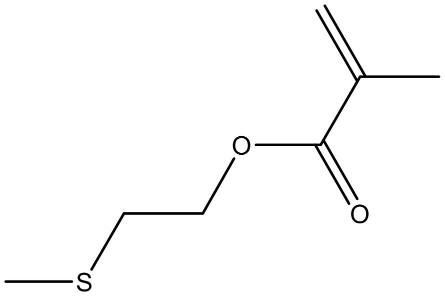
NBMA	Norbornyl methacrylate	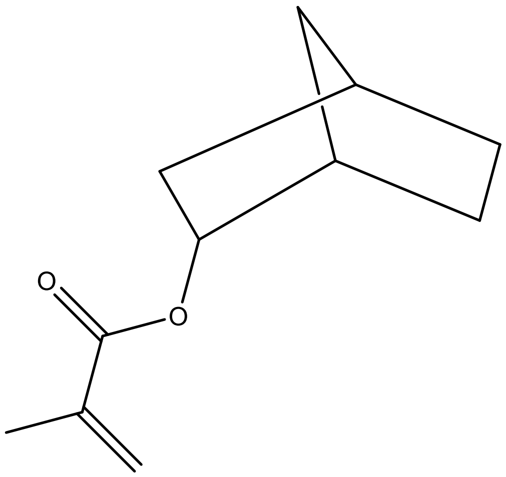
NDMAm	N-Dodecylmethacrylamide	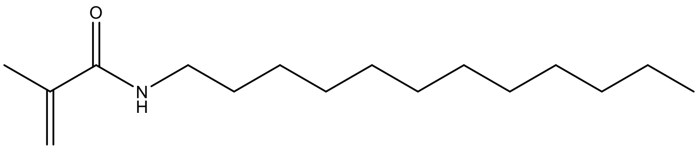

Acronym	Name	Structure
NGDA	Neopentyl glycol diacrylate	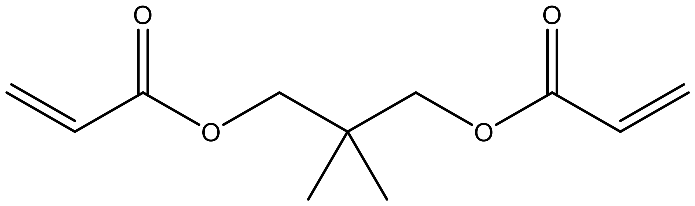
NGPDA	Neopentyl glycol propoxylate diacrylate	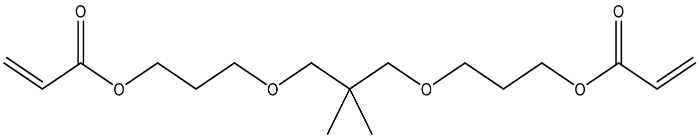
NMEMA	2-N-Morpholinoethyl methacrylate	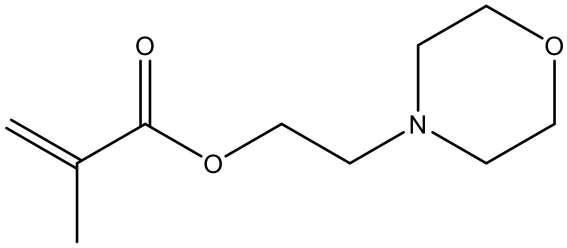
NpA	Naphthyl acrylate	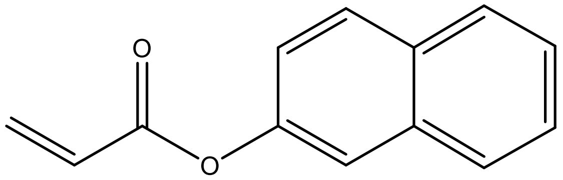
NpMA	Naphthyl methacrylate	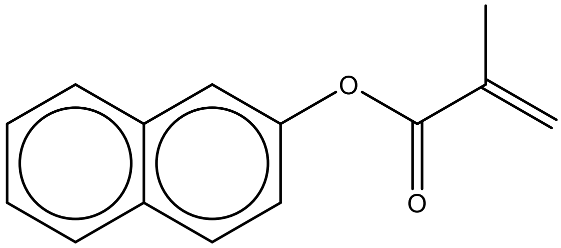
ODA	Octadecyl acrylate	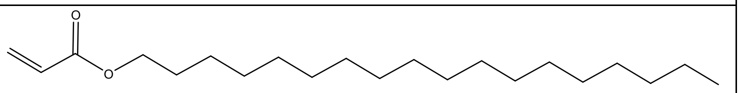
PA	Propargyl acrylate	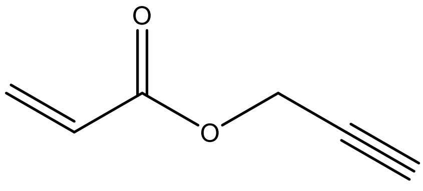

Acronym	Name	Structure
PEDAM	Pentaerythritol diacrylate monostearate	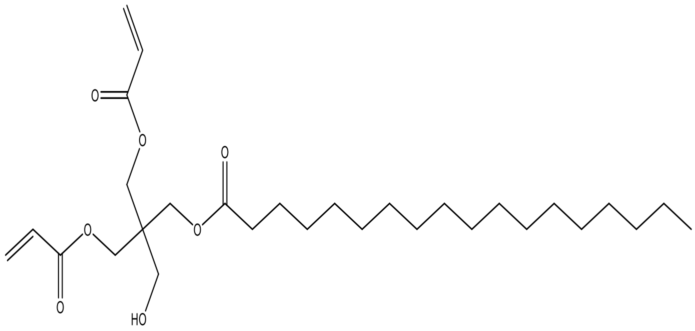
pEGMA	Poly(ethylene glycol) methacrylate	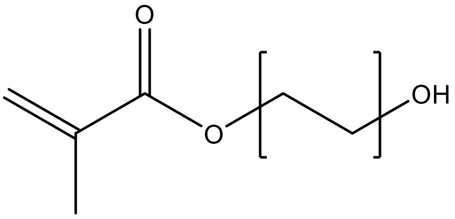
pEGMEMA	Poly(ethylene glycol) methyl ether methacrylate	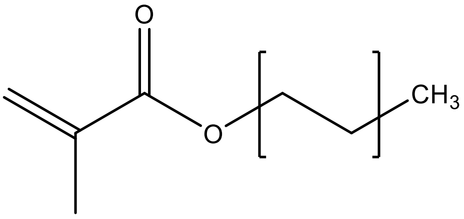
PETA	Pentaerythritol tetraacrylate	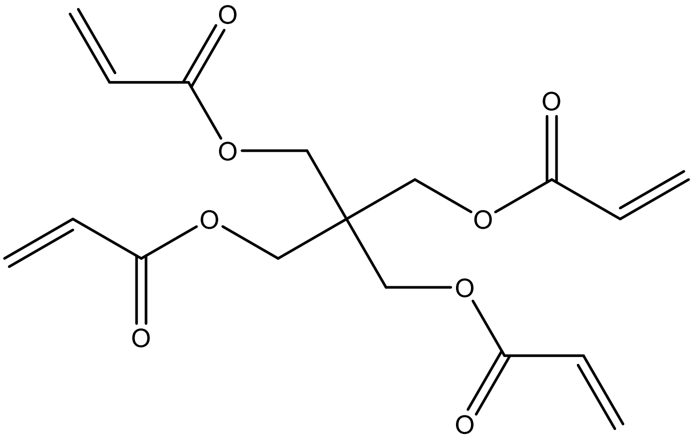
PhMA	Phenyl methacrylate	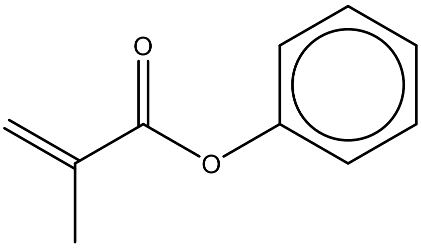

Acronym	Name	Structure
PhMAm	N-Phenylmethacrylamide	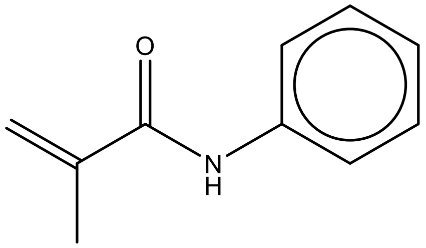
PMAm	N-(Phthalimidomethyl)acrylamide	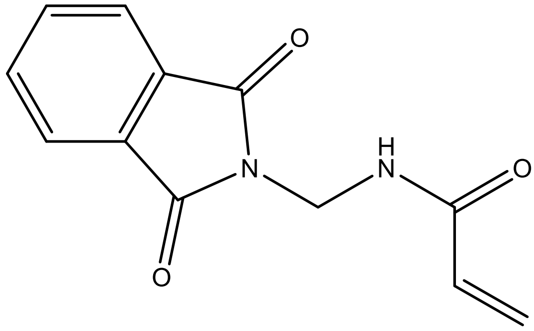
pPGNEA	Poly(propylene glycol) 4-nonylphenyl ether acrylate	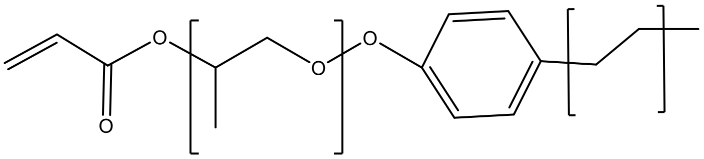
SPAK	Sulfopropyl acrylate potassium salt	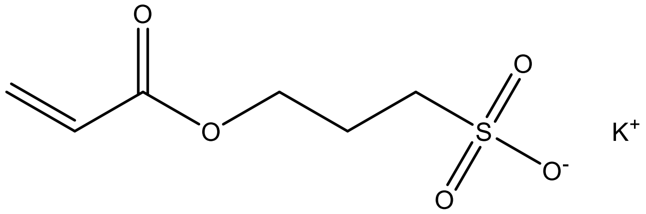
SPMAK	3-Sulfopropyl methacrylate potassium salt	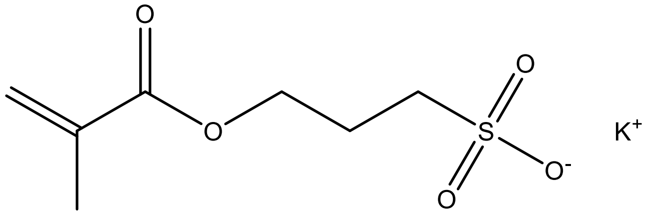

Acronym	Name	Structure
TAHTA	1,3,5-Triacryloylhexahydro-1,3,5-triazine	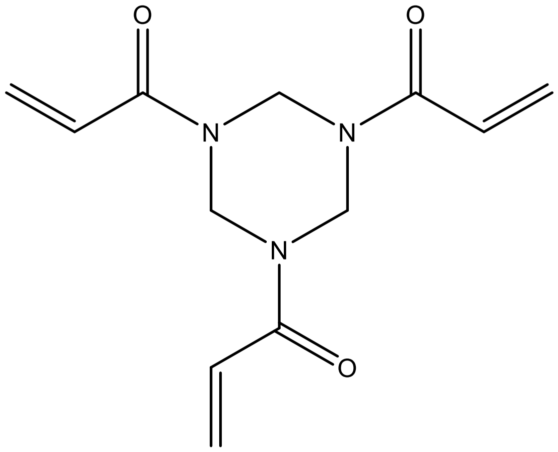
TAIC	Tris[2-(acryloyloxy)ethyl] isocyanurate	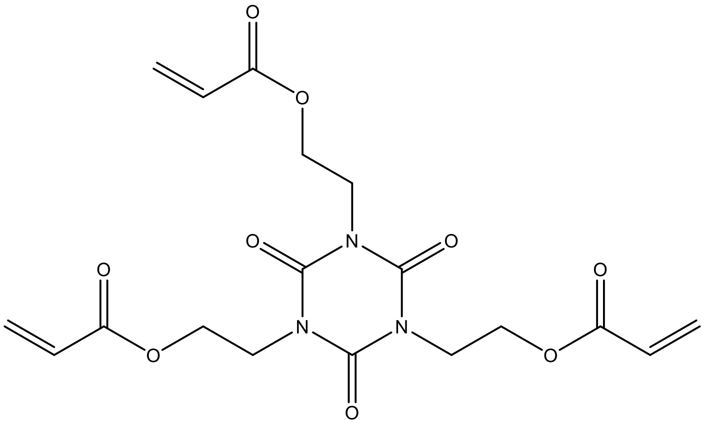
tBA	Tert-butyl acrylate	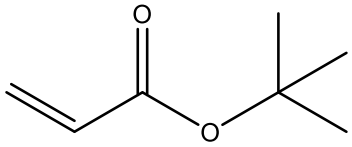
tBAEMA	Tert-butylamino-ethyl methacrylate	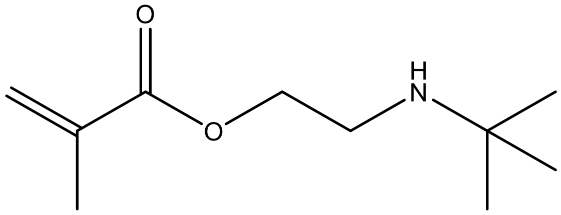
tBAm	N-tert-Butylacrylamide	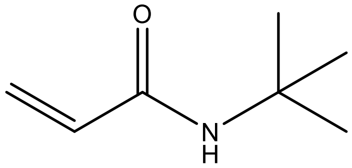

Acronym	Name	Structure
tBCHA	Tert-butylcyclohexylacrylate	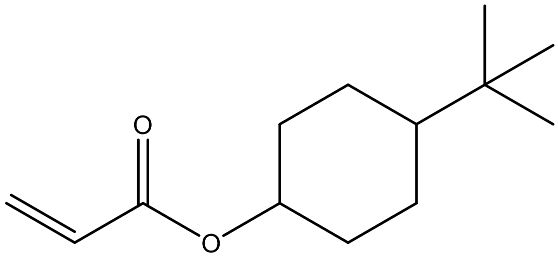
tBCHMA	Tertbutylcyclohexyl methacrylate	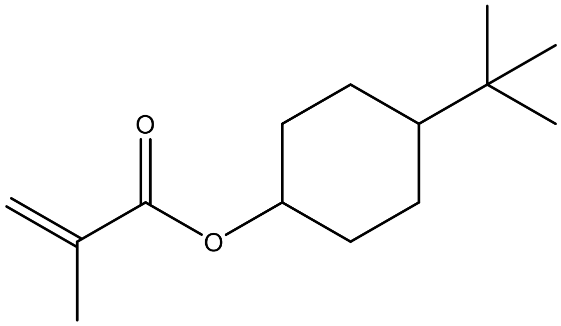
tBMAm	N-tert-Butylmethacrylamide	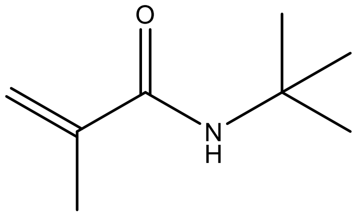
TEGDA	Tetra(ethylene glycol) diacrylate	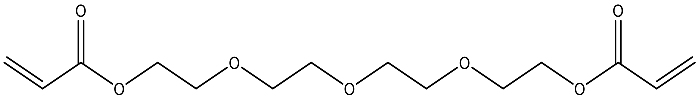
THFuA	Tetrahydrofurfuryl acrylate	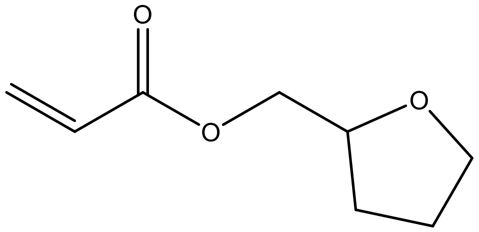
THFuMA	Tetrahydrofurfuryl methacrylate	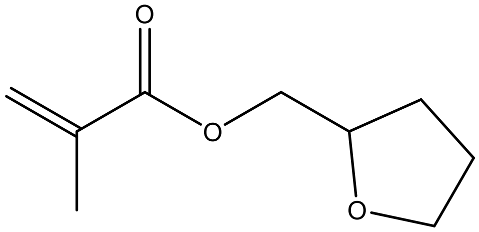

Acronym	Name	Structure
TMCHMA	Trimethylcyclohexyl methacrylate	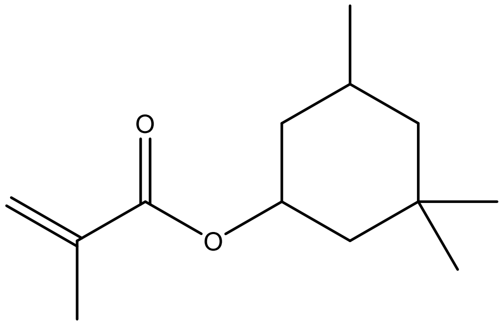
TMHA	Trimethylhexyl acrylate	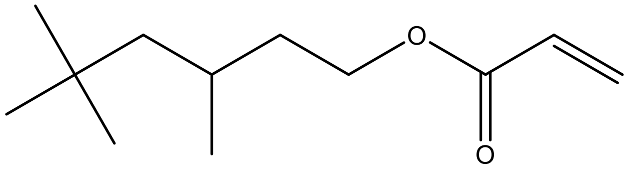
TMPDAE	Trimethyl propane diallyl ether	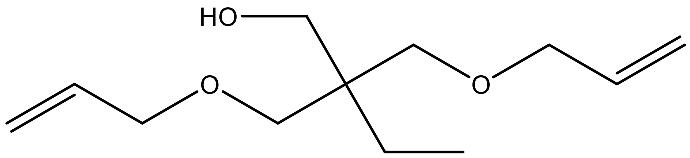
TMPETA	Trimethylolpropane ethoxylate triacrylate	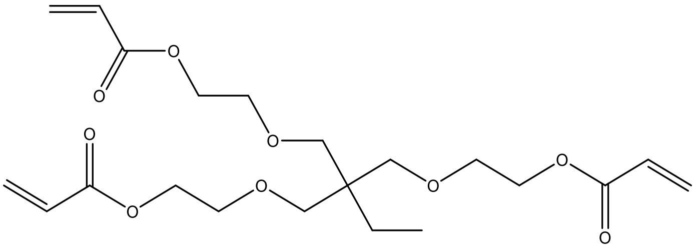
TMPOTA	Trimethylolpropane propoxylate triacrylate	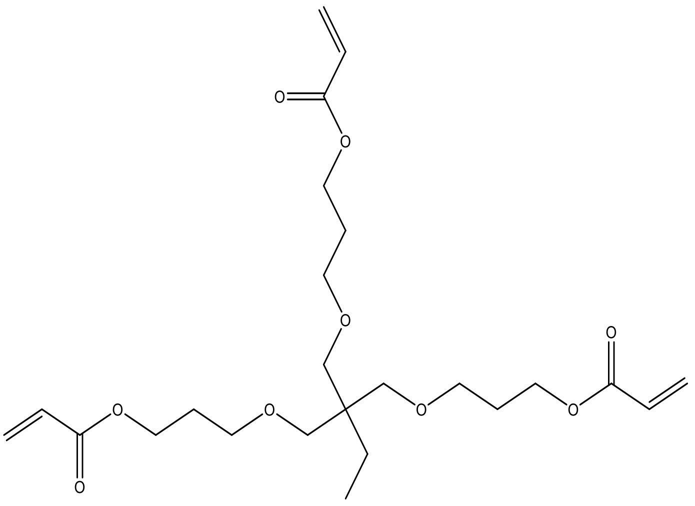

Acronym	Name	Structure
TPGDA	Tri(propylene glycol) diacrylate	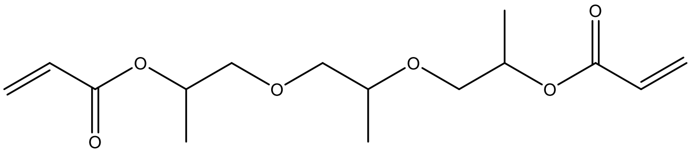
ZnA	Zinc acrylate	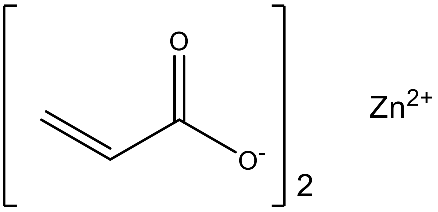
ZrA	Zirconium acrylate	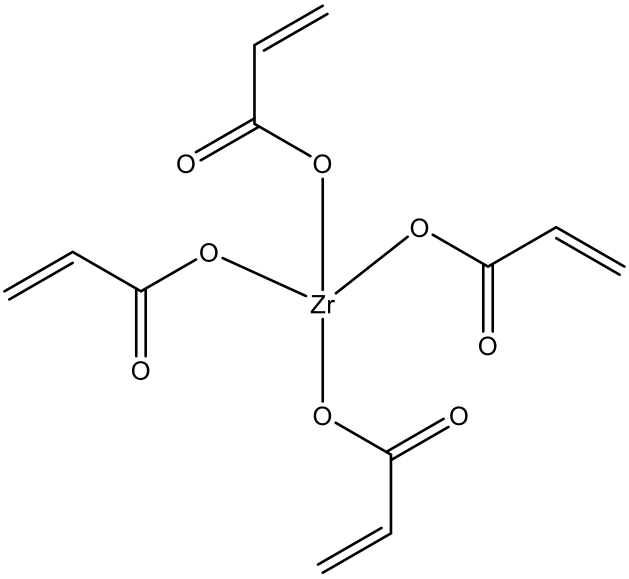
ZrBNCTA	Zirconium bromonorbornanelactone carboxylate triacrylate	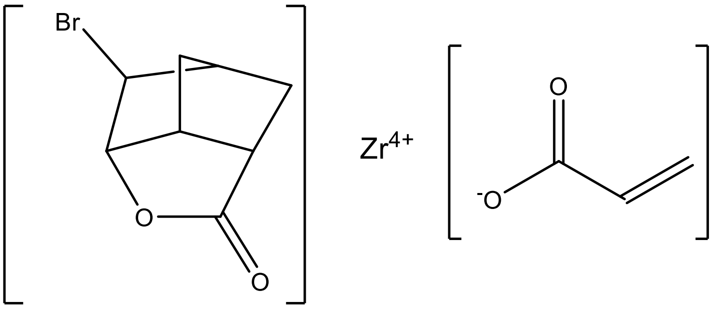
AAm	Acrylamide	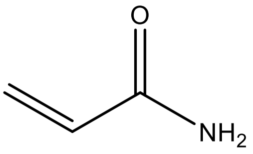

Acronym	Name	Structure
BAC	N,N'-Bis(acryloyl)cystamine	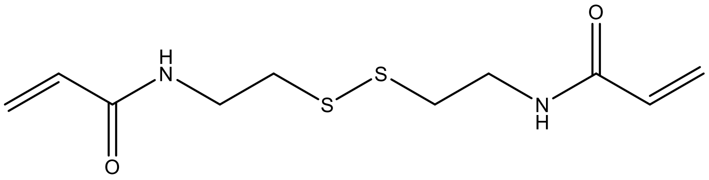
BAPA	1,4-Bis(acryloyl)piperazine	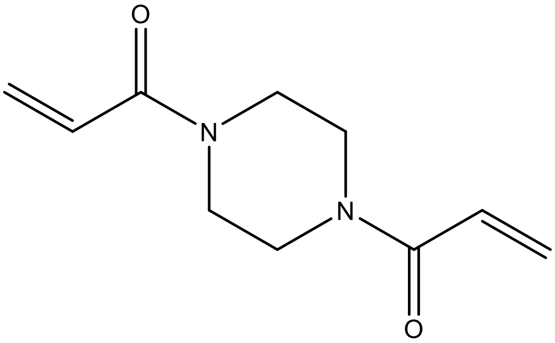
BAPODA	Bisphenol A propoxylate diacrylate	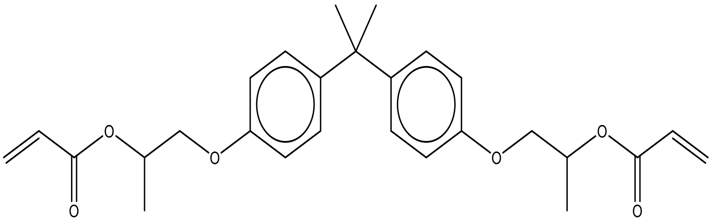
BHMA	Benzhydryl methacrylate	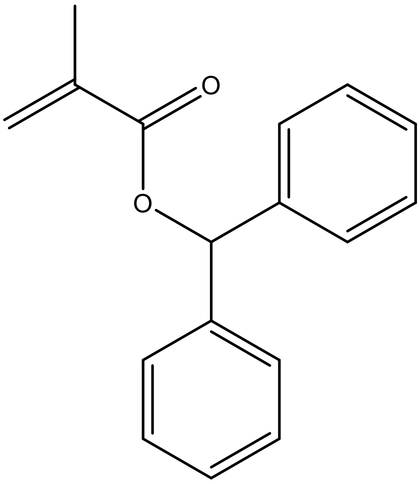
BMAOEP	Bis[2-(methacryloyloxy)ethyl] phosphate	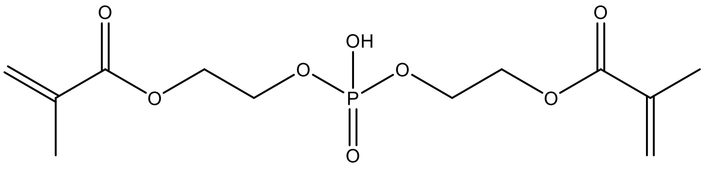

Acronym	Name	Structure
BMENBC	Bis(2-methacryloxyethyl) N,N'-1,9-nonylene biscarbamate	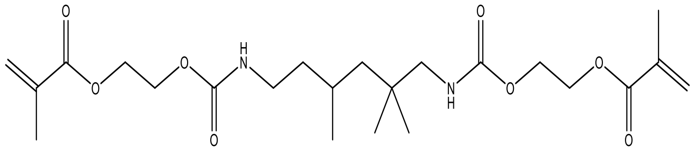
BOEMA	Butoxyethyl methacrylate	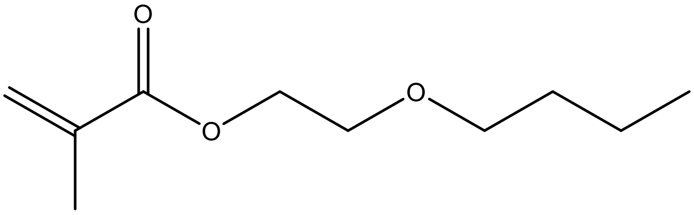
BPDMA	Bisphenol A dimethacrylate	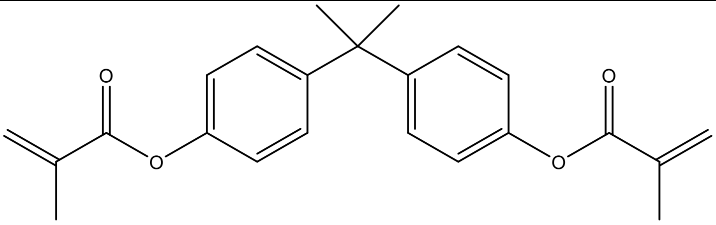
CEA	Carboxyethyl acrylate	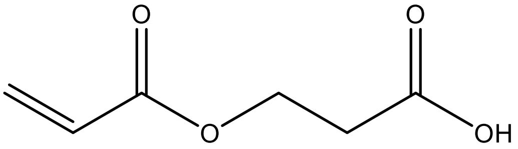
COEA	2-Cinnamoyloxyethyl acrylate	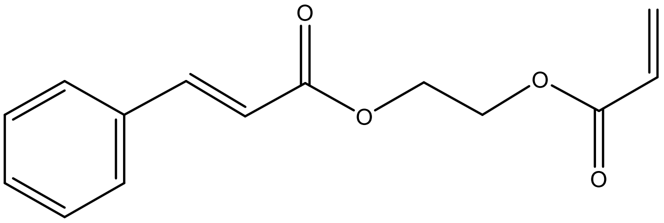

Acronym	Name	Structure
CzEA	Carbazol-9-yl ethyl acrylate	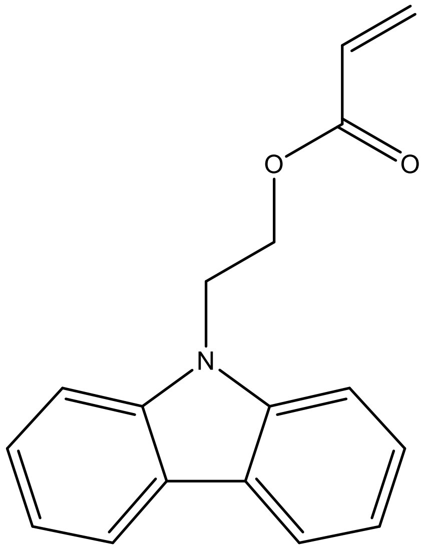
DFFMOA	Dodecafluoro-7-(trifluoromethyl)-octyl acrylate	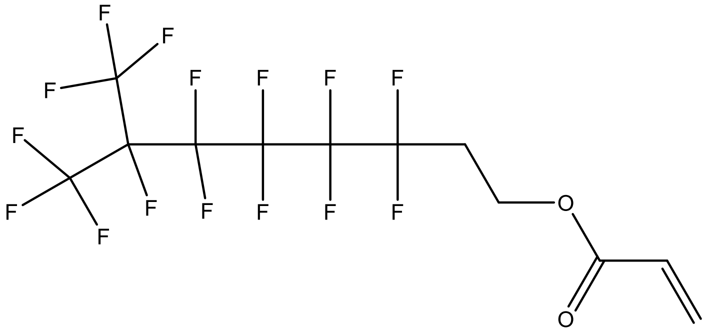
DFHNMA	Dodecafluoro-2-hydroxy-8-(trifluoromethyl)nonyl methacrylate	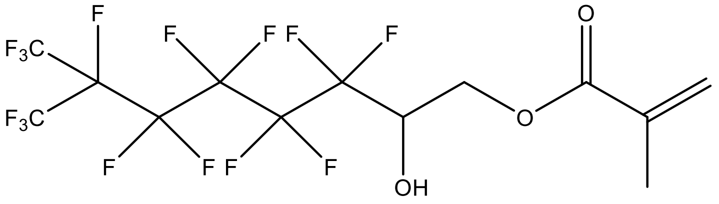
DHEBAM	N,N'-(1,2-Dihydroxyethylene)bisacrylamide	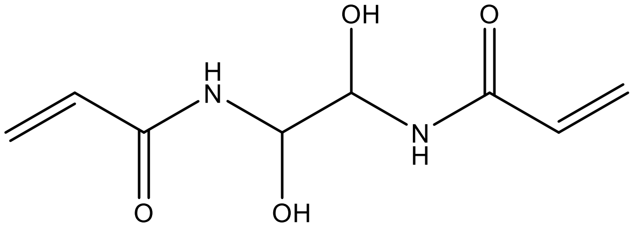

Acronym	Name	Structure
DMEMAm	N-[2-(N,N-Dimethylamino)ethyl]methacrylamide	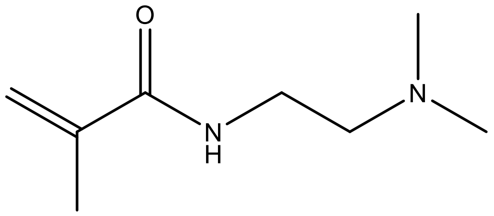
DMMAm	N,N-Dimethylmethacrylamide	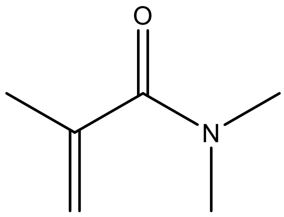
DMPAm	N-[3-(Dimethylamino)propyl]acrylamide	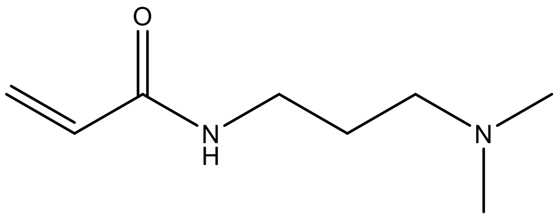
DVAd	Divinyl Adipate	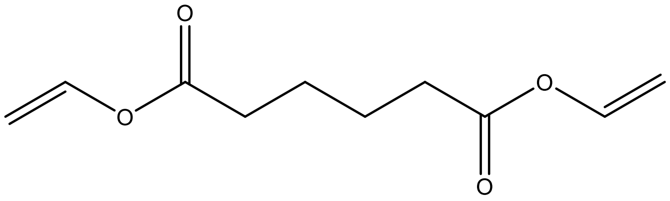
DVSeb	Divinyl sebacate	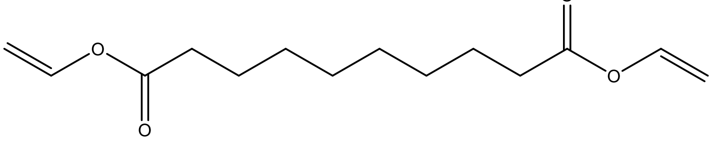
F7BA	Heptafluorobutyl acrylate	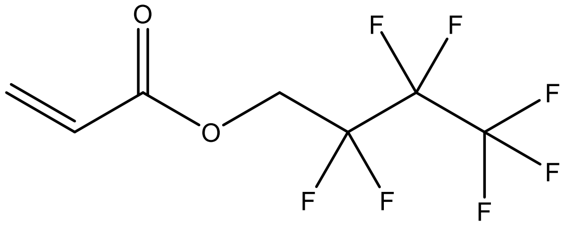

Acronym	Name	Structure
F7BMA	Heptafluorobutyl methacrylate	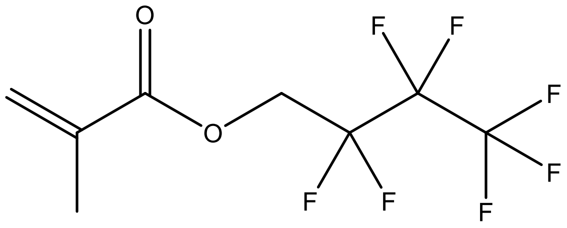
FuMA	Furfuryl methacrylate	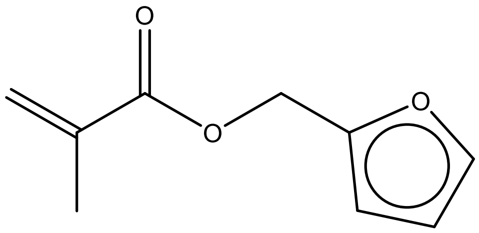
GDGDA	Glycerol 1,3-diglycerolate diacrylate	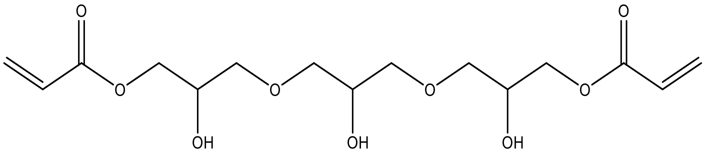
GDMA	Glycerol dimethacrylate	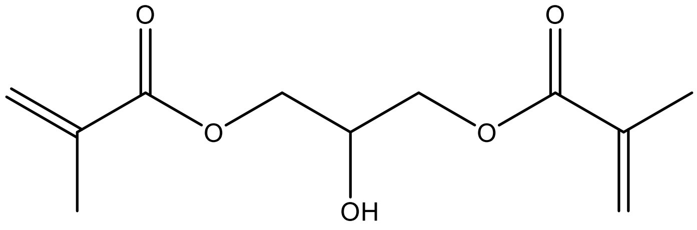
HBMA	Hydroxybutyl methacrylate	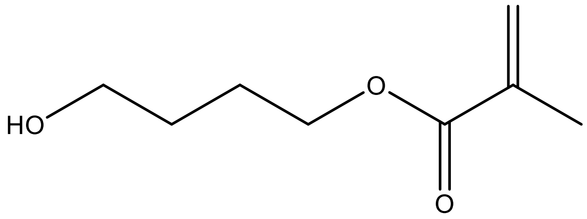
HDFDA	Heptadecafluorodecyl acrylate	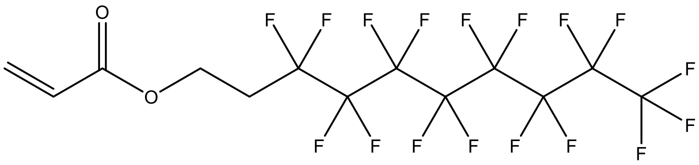

Acronym	Name	Structure
HDFDMA	Heptadecafluorodecyl methacrylate	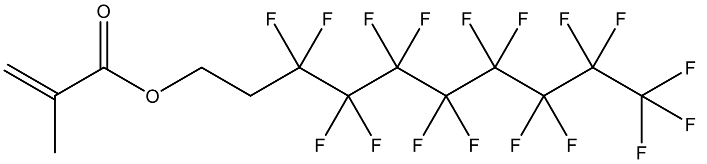
HDMA	1-Hexadecyl methacrylate	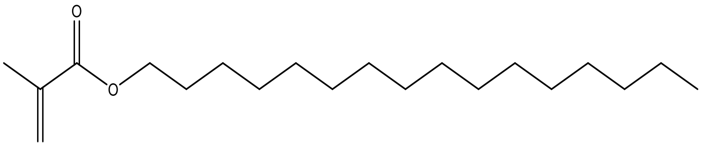
HEODA	Hexanediol ethoxylate diacrylate	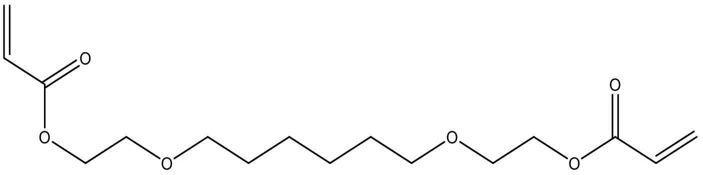
HFDA	Heneicosafluorododecyl acrylate	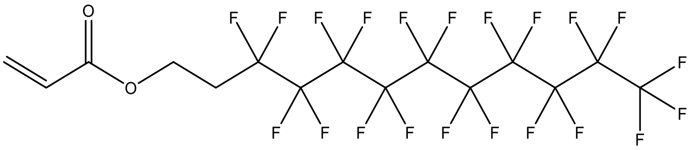
HFHUMA	Hexadecafluoro-2-hydroxy-10-(trifluoromethyl)undecyl methacrylate	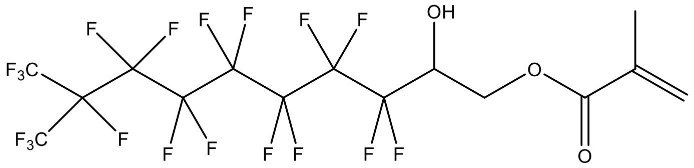
HMAm	N-(Hydroxymethyl)acrylamide	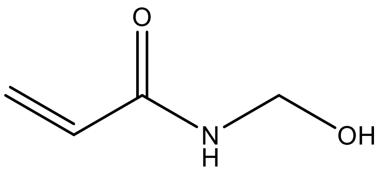
HMBMAm	N,N'-Hexamethylenebis(methacrylamide)	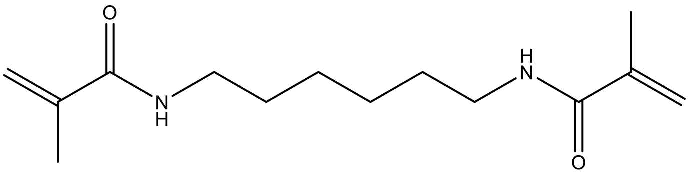

Acronym	Name	Structure
HPMAm	N-(2-Hydroxypropyl)methacrylamide	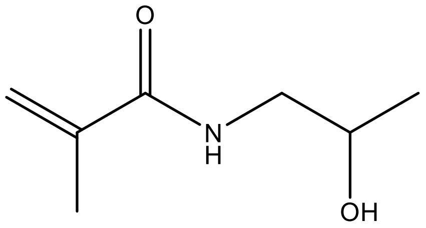
iCEMA	Isocyanatoethyl methacrylate	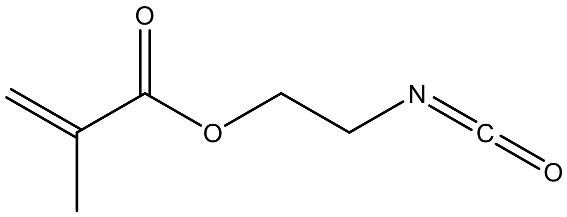
MA	Methyl acrylate	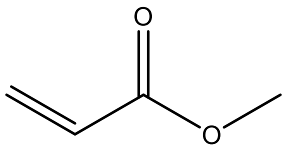
MAAHS	Methacrylic acid N-hydroxysuccinimide ester	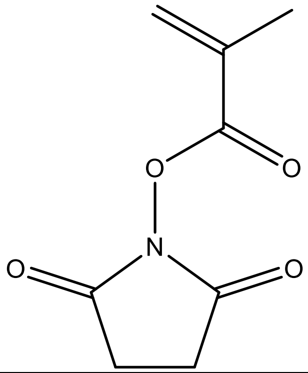
MAETA	4-Methacryloxyethyl trimellitic anhydride	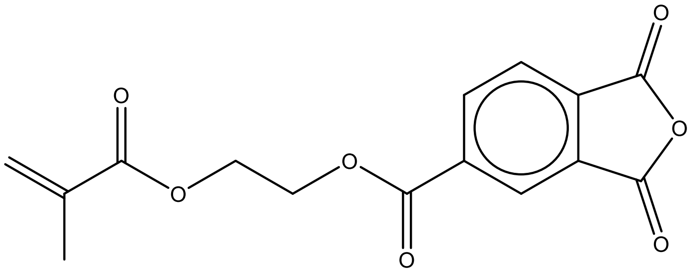
MAPU	2-methacryloxyethyl phenyl urethane	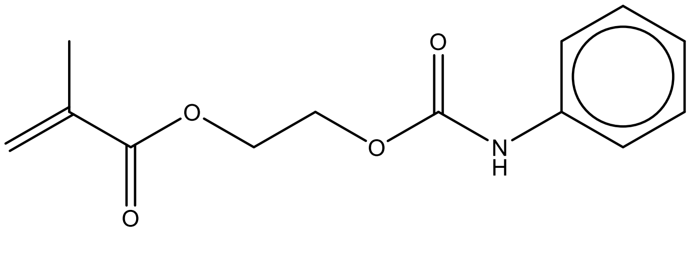

Acronym	Name	Structure
MHMB	Methyl 3-hydroxy-2-methylenebutyrate	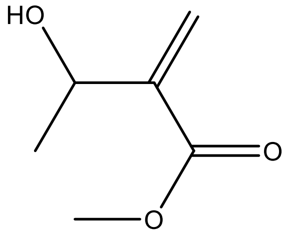
MMA	Methyl methacrylate	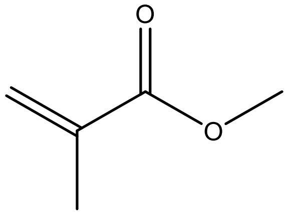
mMAOEM	mono-2-(Methacryloyloxy)ethyl maleate	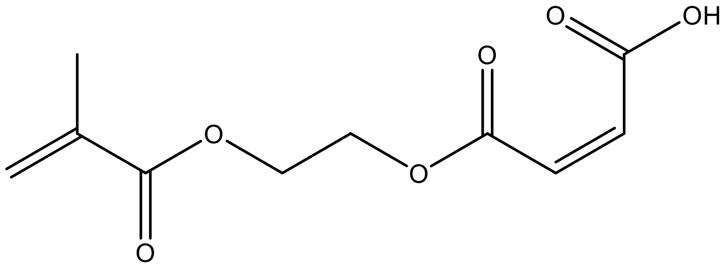
mMAOES	mono-2-(Methacryloyloxy)ethyl succinate	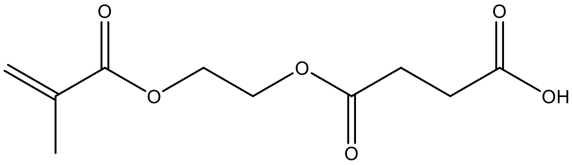
MOPAm	*N*-(3-Methoxypropyl)acrylamide	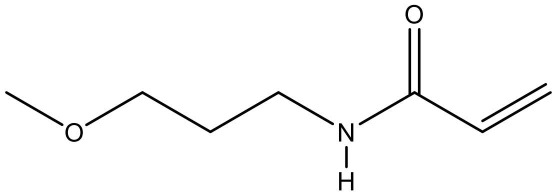
NAM	*N*-Acryloylmorpholine	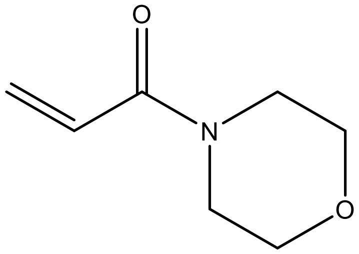

Acronym	Name	Structure
NAS	*N*-Acryloxysuccinimide	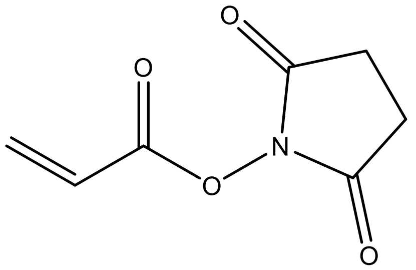
NBnMA	*o*-Nitrobenzyl methacrylate	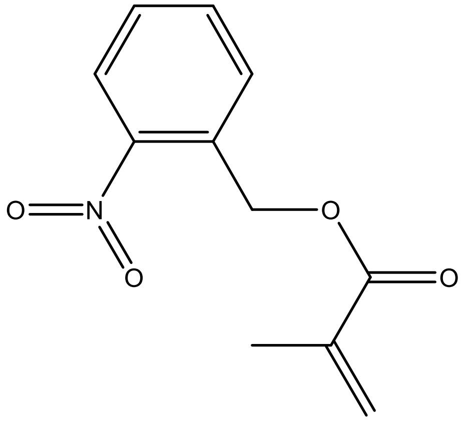
NDDMA	1,9-Nonanediol dimethacrylate	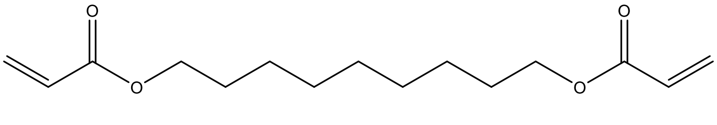
nOcMA	n-Octyl methacrylate	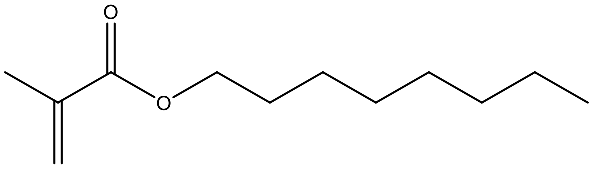
NPhPMA	Nitrophenyl-2-pyrrolidonemethyl acrylate	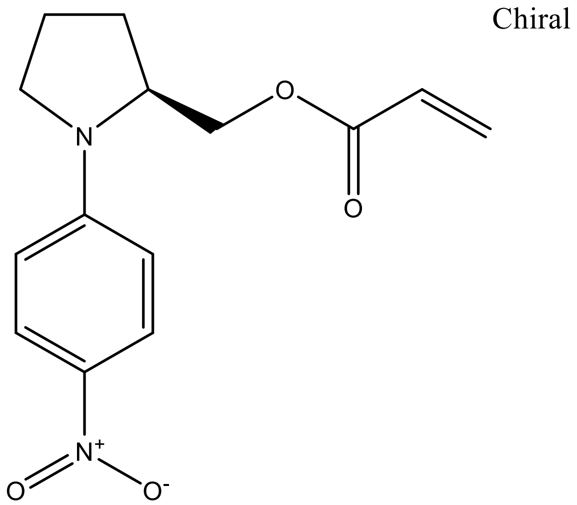

Acronym	Name	Structure
OFHMA	Octafluoro-2-hydroxy-6-(trifluoromethyl)heptyl methacrylate	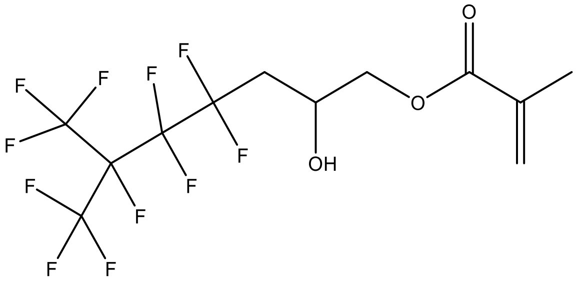
OFPA	Octafluoropentyl acrylate	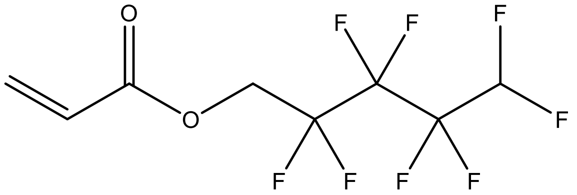
PAHEMA	Phosphoric acid 2-hydroxyethyl methacrylate ester	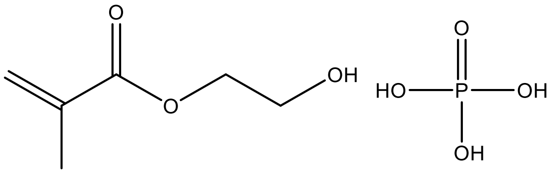
PBBA	Pentabromobenzyl acrylate	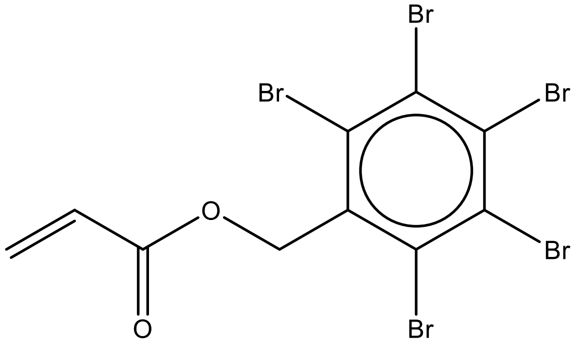
PDDMA	1,5-Pentanediol dimethacrylate	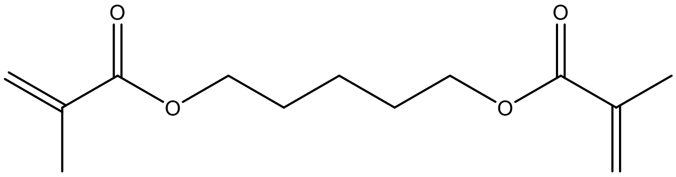

Acronym	Name	Structure
pEGDA	Polyethylene glycol diacrylate	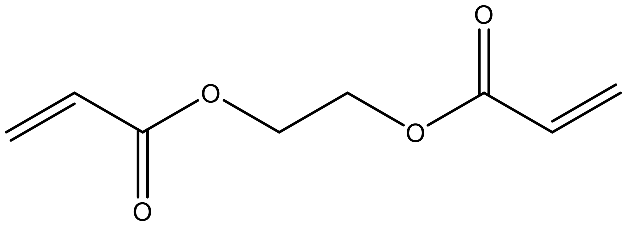
PETrA	Pentaerythritol triacrylate	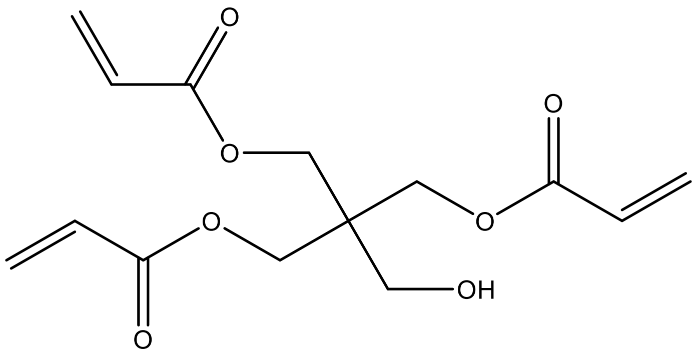
pFDA	Perfluorodecyl acrylate	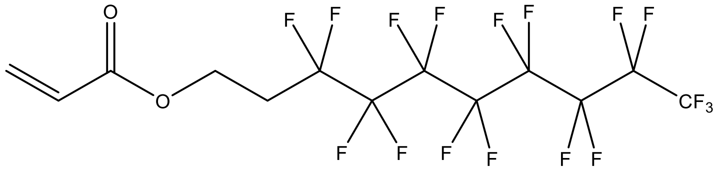
PFPA	Pentafluoropropyl acrylate	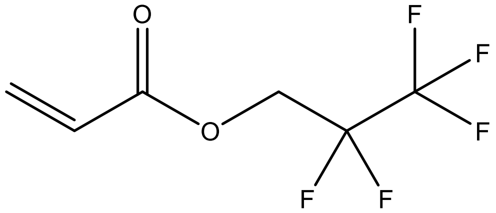
PFPMA	Pentafluoropropyl methacrylate	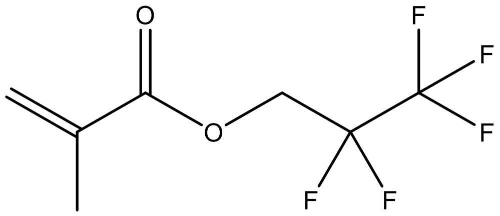
PhA	Phenyl acrylate	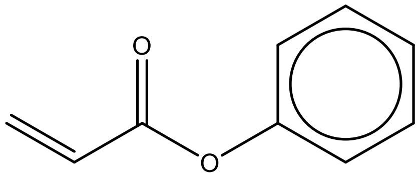

Acronym	Name	Structure
PMA	Propargyl methacrylate	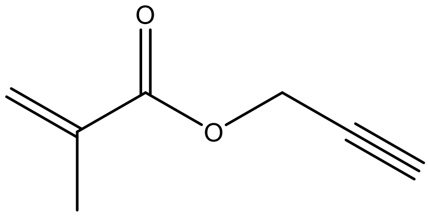
PMMA	1-Pyrenylmethyl methacrylate	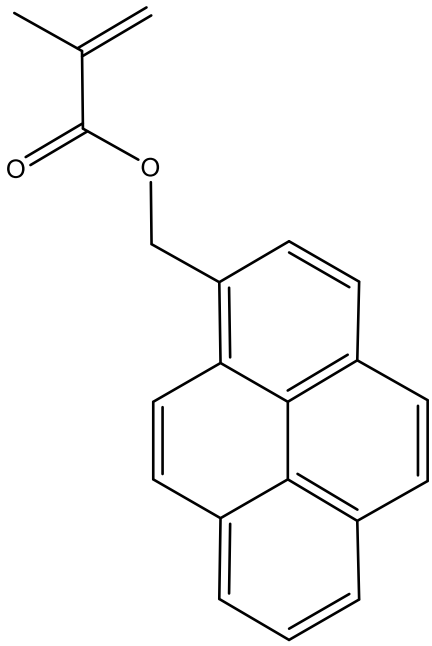
pPGDA	Poly(propylene glycol) diacrylate	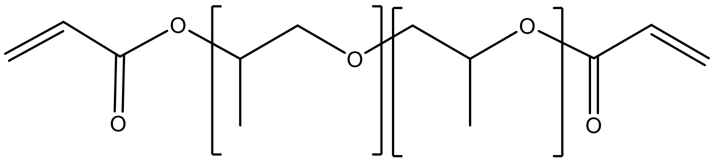
SEMA	2-Sulfoethyl methacrylate	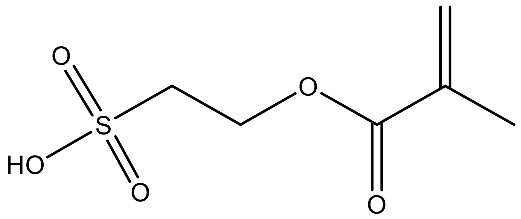
tBMA	Tert-butyl methacrylate	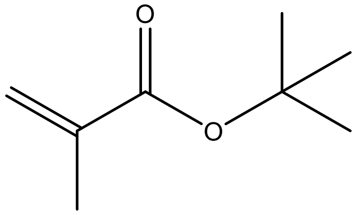

Acronym	Name	Structure
TBNpMA	Tribromoneopentyl methacrylate	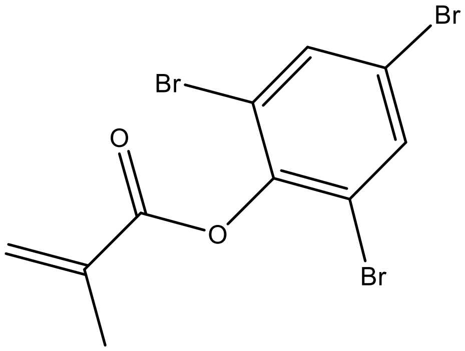
TBPhA	2,4,6-Tribromophenyl acrylate	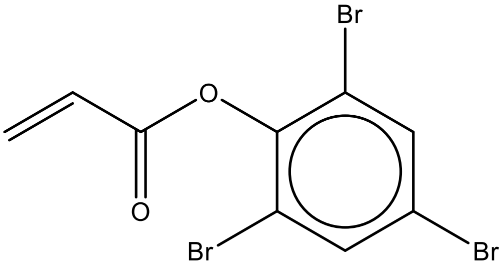
TBPMA	Tribromophenyl methacrylate	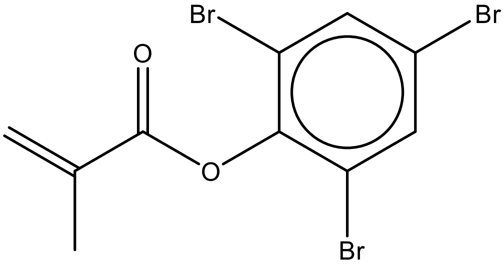
TCDMDA	Tricyclodecane-dimethanol diacrylate	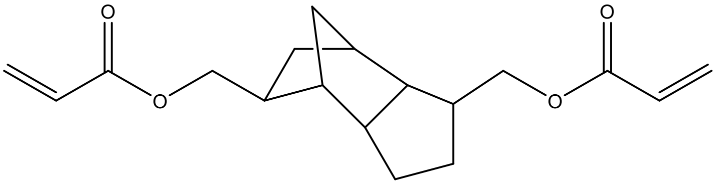
TDFOcA	Tridecafluorooctyl acrylate	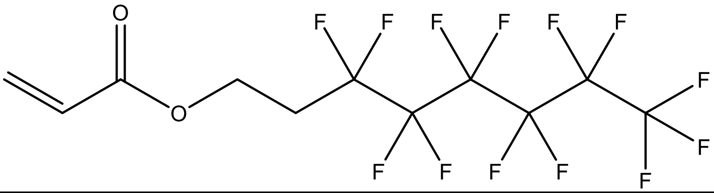
TEGMA	Tri(ethylene glycol) methyl ether methacrylate	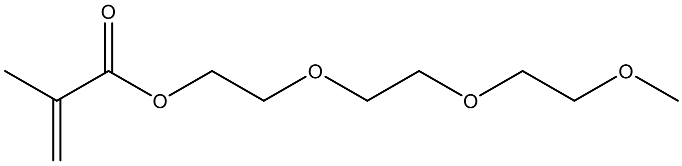

Acronym	Name	Structure
TFPMA	Tetrafluoropropyl methacrylate	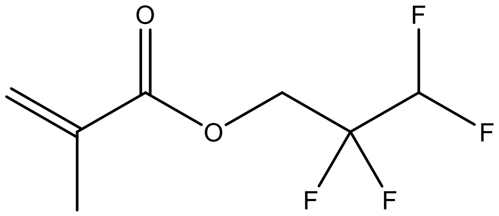
VMA	Vinyl methacrylate	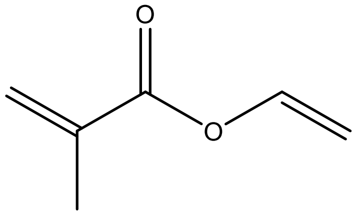
ZrCEA	Zirconium carboxyethyl acrylate	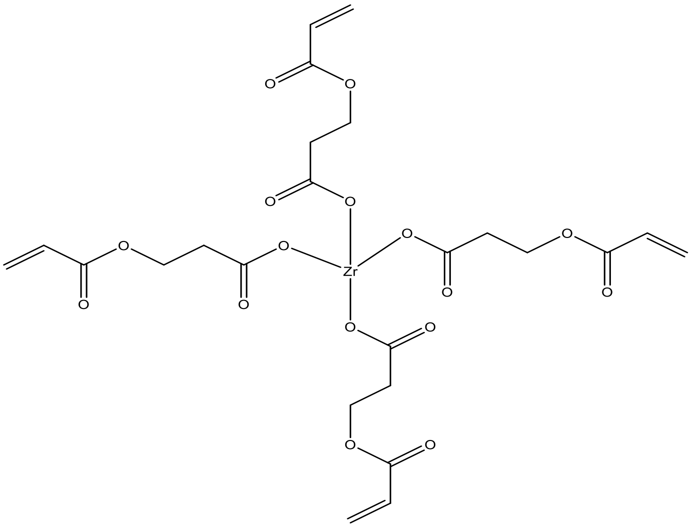

Acronym	Name	Structure
DFHA	Dodecafluoroheptyl acrylate	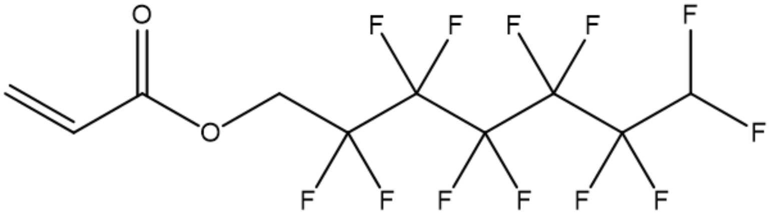
EHMA	Ethylhexyl methacrylate	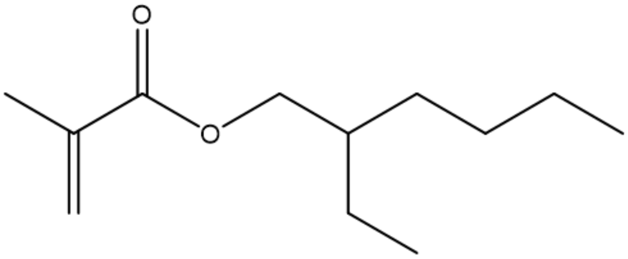
DYA	Disperse yellow 7 acrylate	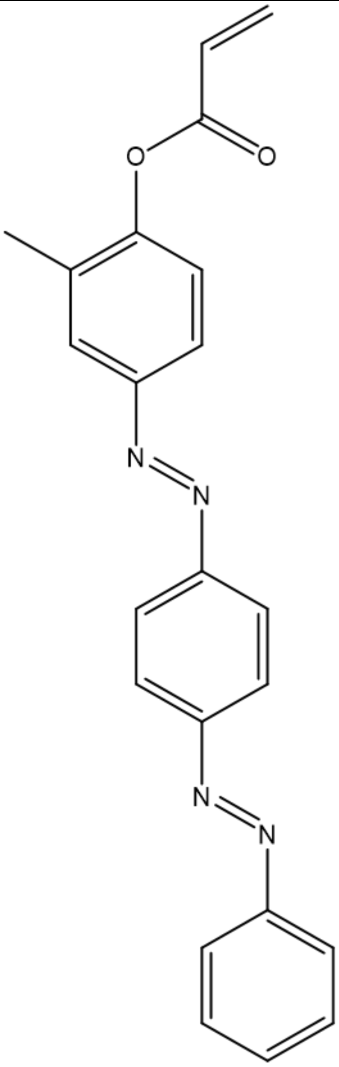

Acronym	Name	Structure
BTHPhMA	Benzotriazol-2-yl)-4-hydroxyphenyl]ethyl methacrylate	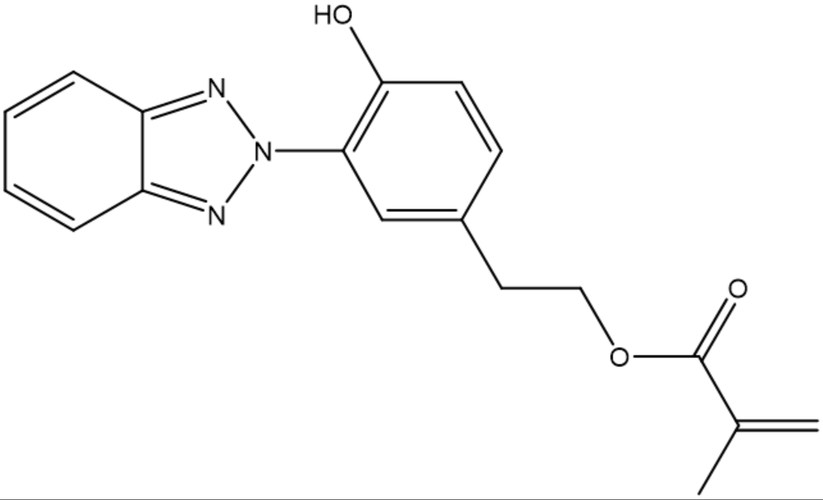
CHPMA	Chloro-2-hydroxy-propyl methacrylate	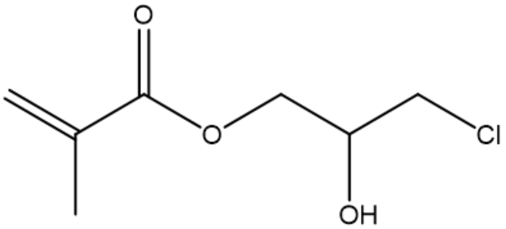
14BDDMA	1,4-Butanediol dimethacrylate	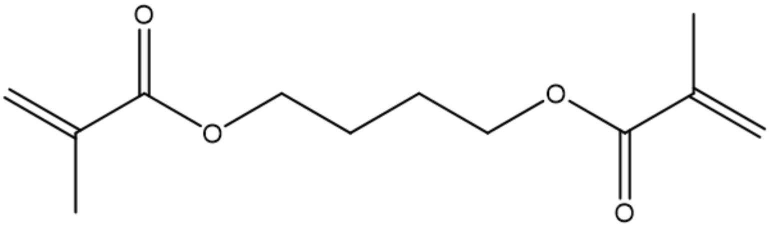
CHA	Cyclohexyl acrylate	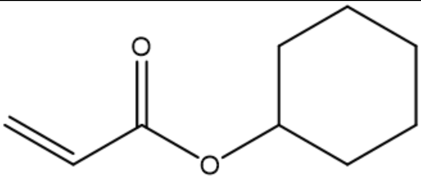
DMAPA	Dimethylamino-propyl acrylate	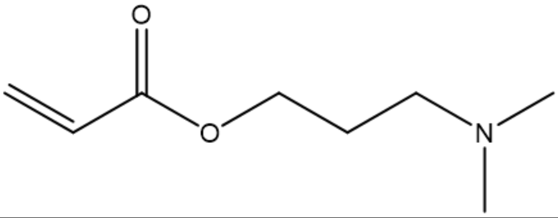
DEGDA	Di(ethylene glycol) diacrylate	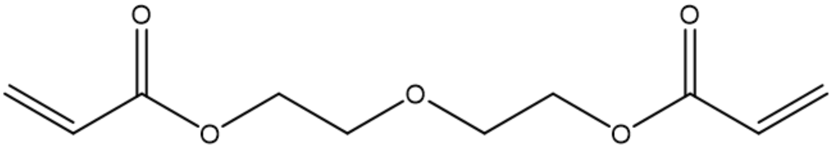

Acronym	Name	Structure
F6BA	Hexafluorobutyl acrylate	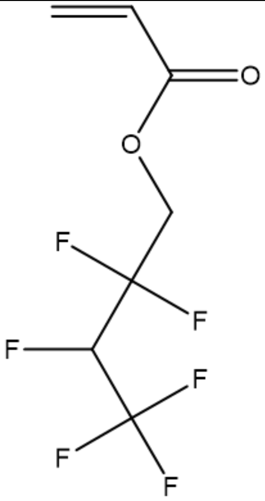
AEMA.C	2-Aminoethyl methacrylate hydrochloride	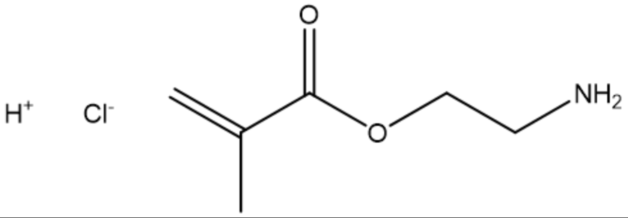
DAAM	N,N-Diallylacrylamide	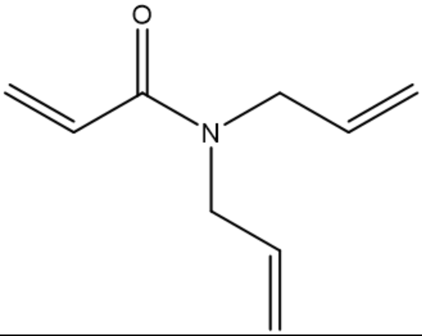
DMA	Decyl methacrylate	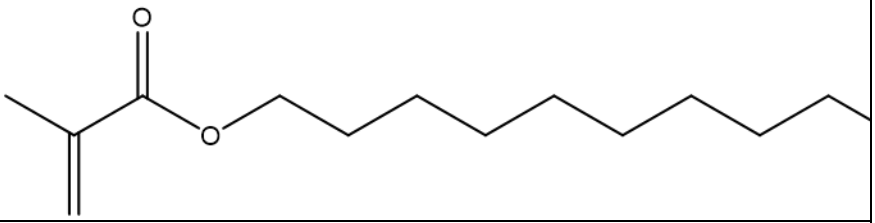
EGDA	Ethylene glycol diacrylate	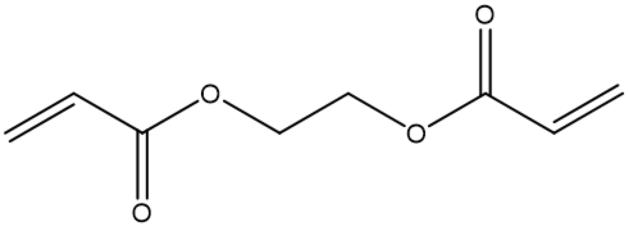

Acronym	Name	Structure
DOAm	Disperse Orange 3 acrylamide	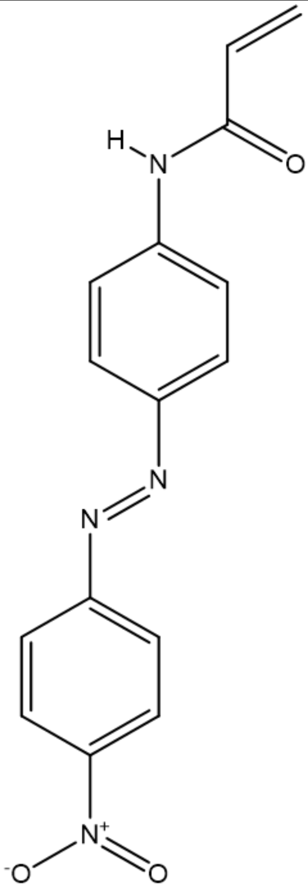
BMAM	N-Benzylmethacrylamide	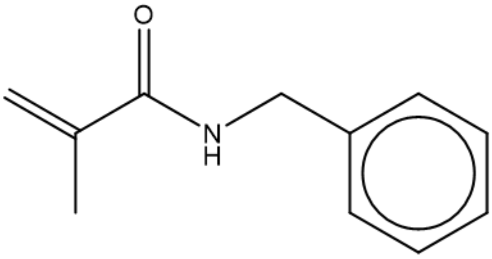
DEAEA	Diethylamino ethyl acrylate	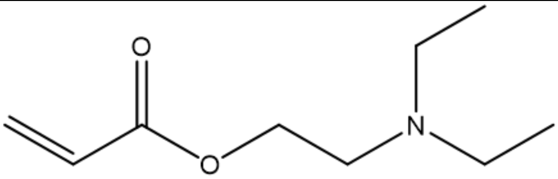
AEMAm.C	N-(2-aminoethyl) methacrylamide hydrochloride	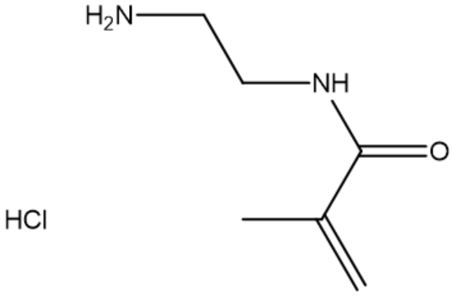

Acronym	Name	Structure
BMA	Butyl methacrylate	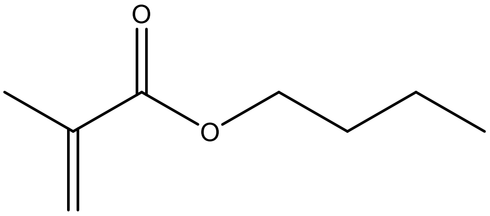
BA	Butyl acrylate	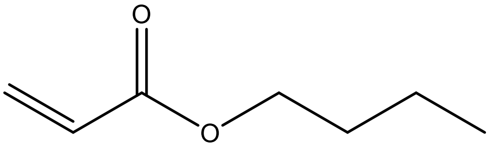
EBAM	N,N'-Ethylenebisacrylamide	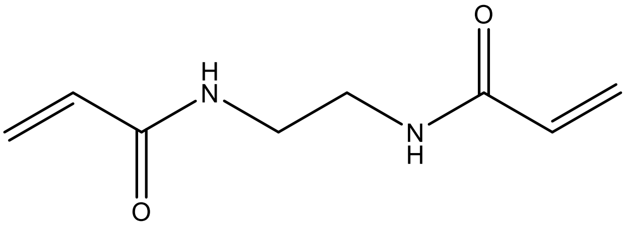
HBA	Hydroxybutyl acrylate	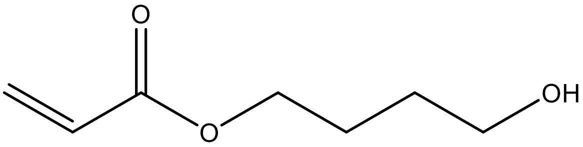
DiPEMA	2-Diisopropylaminoethyl methacrylate	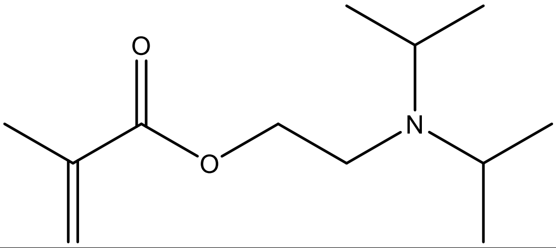
HFPDA	Hexafluoropent-1,5-diyl diacrylate	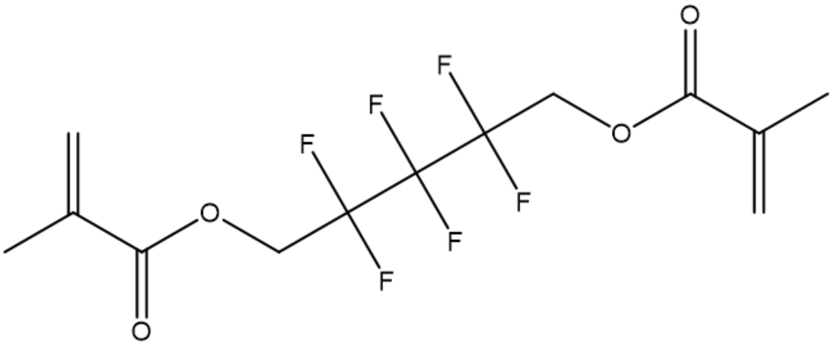
HPhOPA	Hydroxy-3-phenoxypropyl acrylate	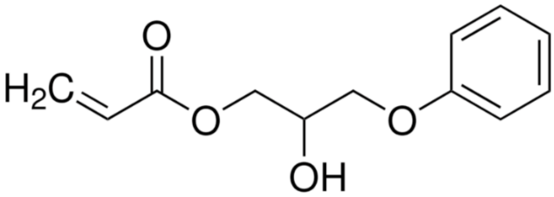
DMAm	N,N'-Dimethylacrylamide	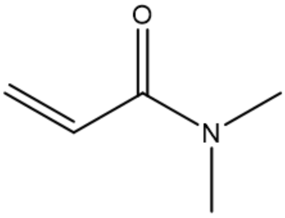

Acronym	Name	Structure
BHMA	Benzhydryl methacrylate	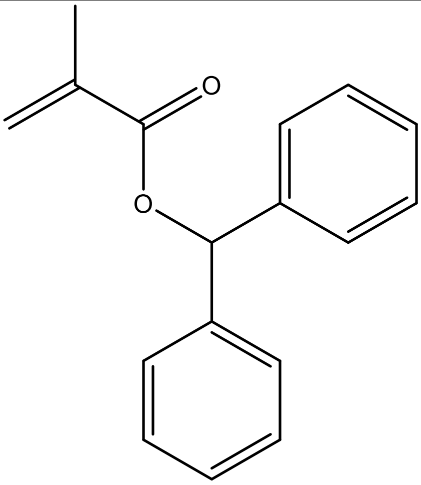
CNEA	Cyanoethyl acrylate	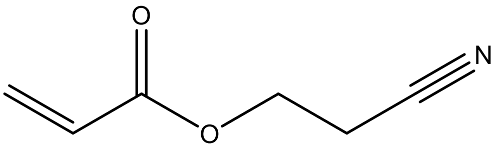
HMBMAm	N,N'-Hexamethylenebis (methacrylamide)	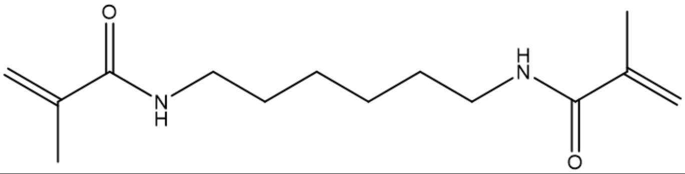
HDFDMA	Heptadecafluorodecyl methacrylate	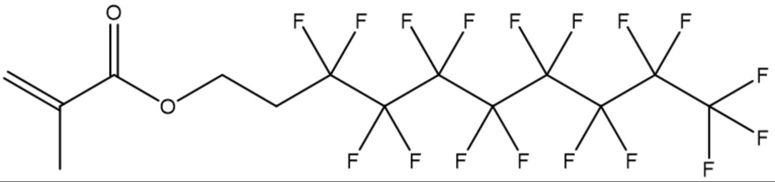
iCEMA	Isocyanatoethyl methacrylate	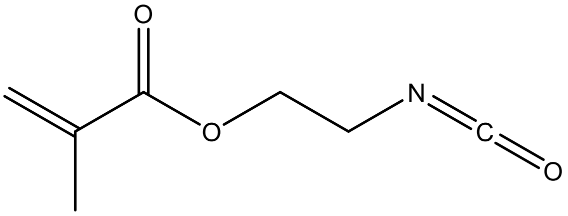
pEGMEA	Poly(ethylene glycol) methyl ether acrylate	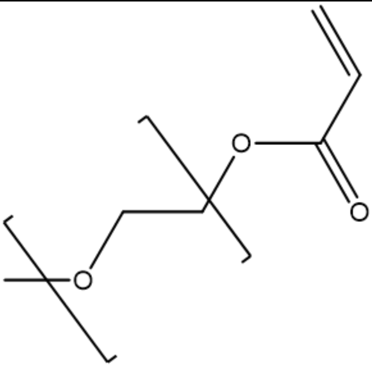
pPGDA	Poly(propylene glycol) diacrylate	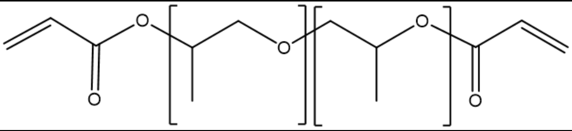

Acronym	Name	Structure
PHPMA	3-Phenoxy 2 hydroxy propyl methacrylate	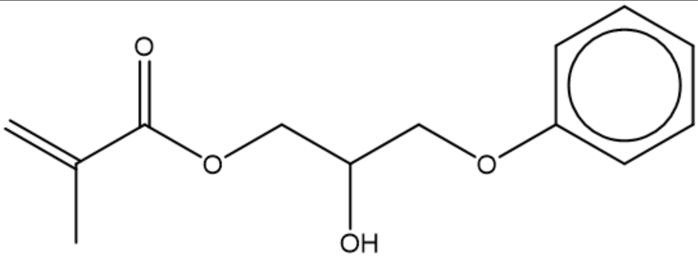
PhEA	2-Phenylethyl acrylate	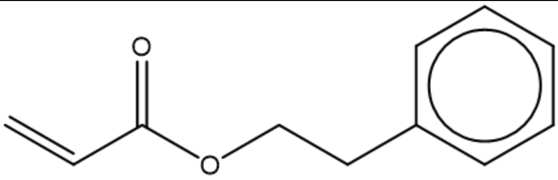
TDFOMA	Tridecafluorooctyl methacrylate	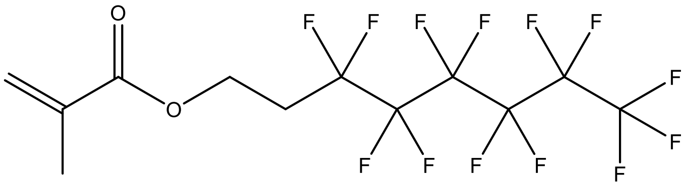
BnA	Benzyl acrylate	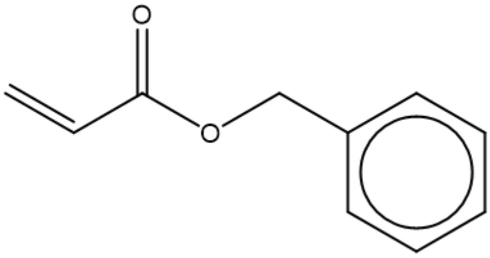
TFCAm	7-[4-(Trifluoromethyl)coumarin]acrylamide	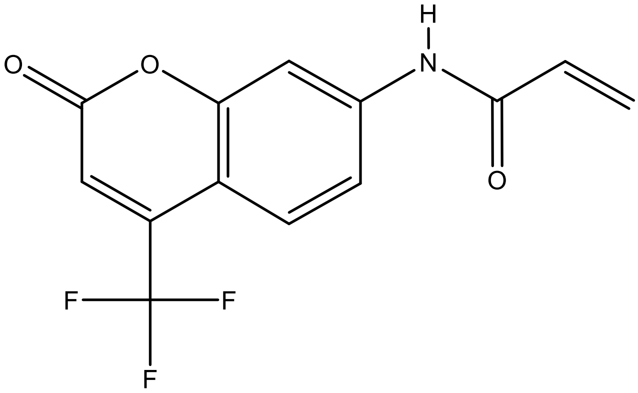
TPhMAm	N-(Triphenylmethyl) methacrylamide	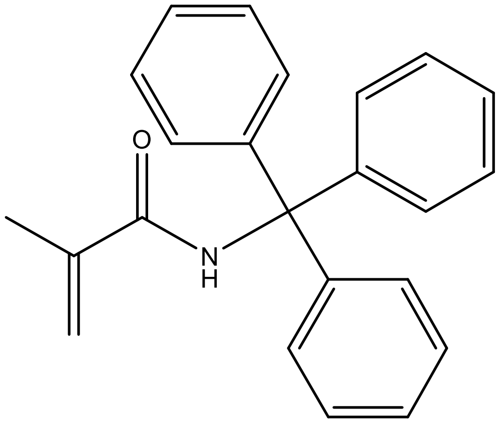

Acronym	Name	Structure
THMMAm	N-[Tris(hydroxymethyl)methyl]acrylamide	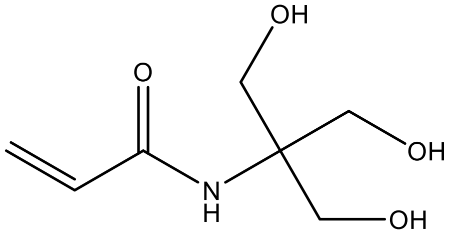
EA	Ethyl acrylate	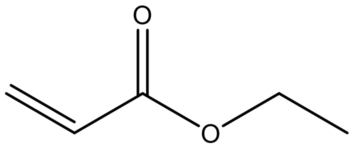
EGMMA	Ethylene glycol methyl ether methacrylate	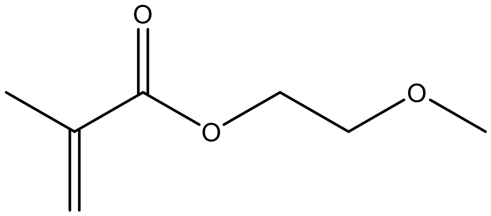
EGPhMA	Ethylene glycol phenyl ether methacrylate	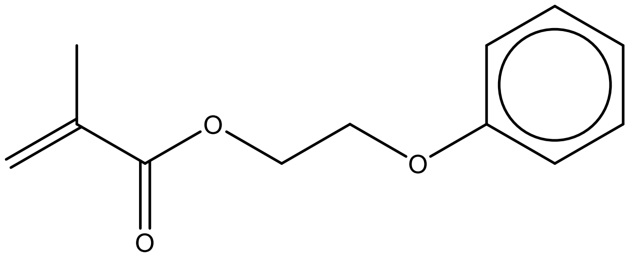
E3GDA	Triethylene glycol diacrylate	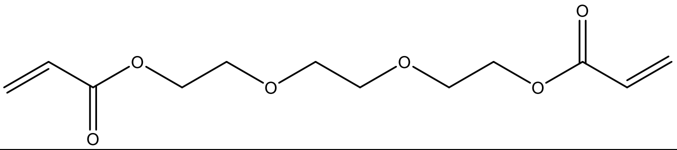
HDMPDA	Hydroxy-2,2-dimethylpropyl 3-hydroxy-2,2-dimethylpropionate diacrylate	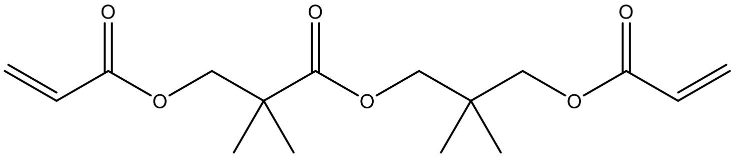

Acronym	Name	Structure
HfCEA	Hafnium carboxyethyl acrylate	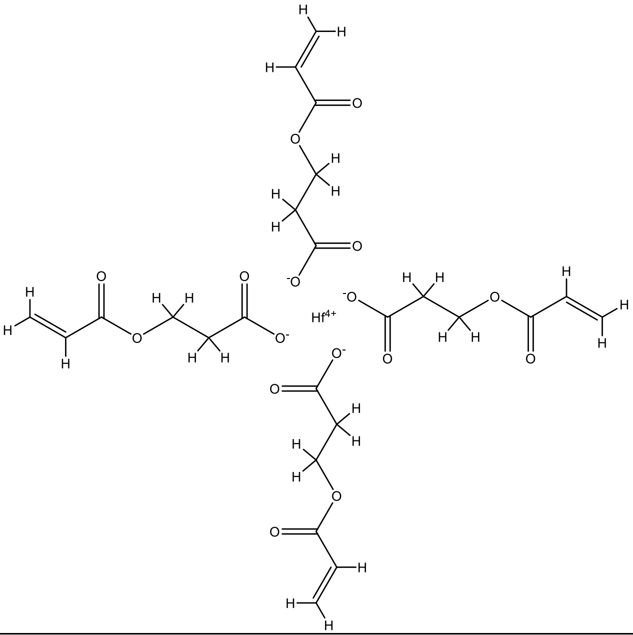
EbCNA	Ethyl-cis-B-cyano-acrylate	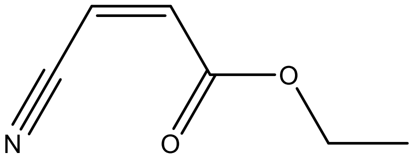
iDMA	Isodecyl methacrylate	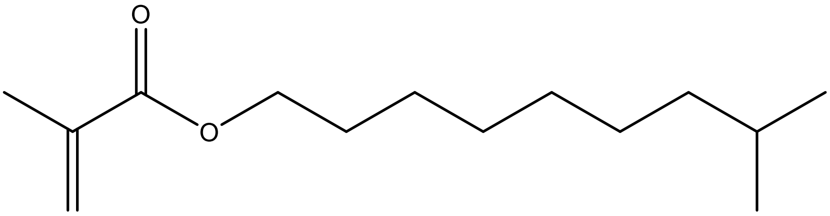
HA	Hexyl acrylate	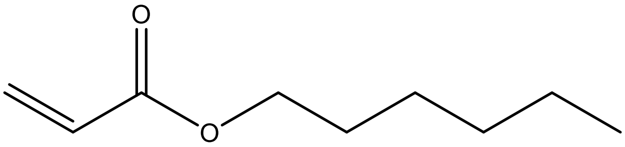
GPOTA	Glycerol propoxylate triacrylate	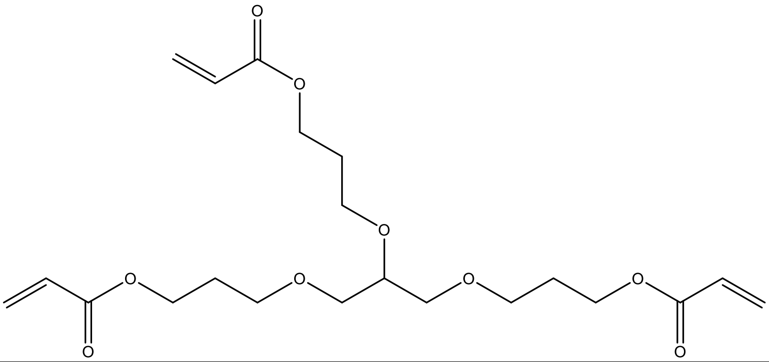
MAEACl	[2-(Methacryloyloxy) ethyl]trimethylam monium chloride solution	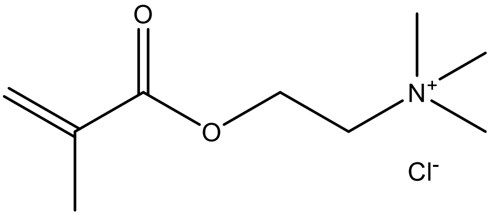

Acronym	Name	Structure
MAHBP	4-Methacryloxy-2-hydroxybenzophenone	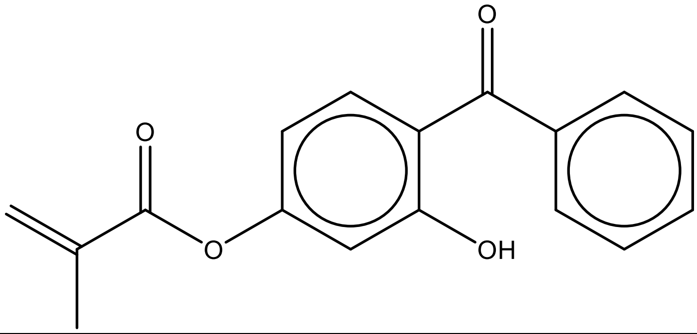
OFPMA	Octafluoropentyl methacrylate	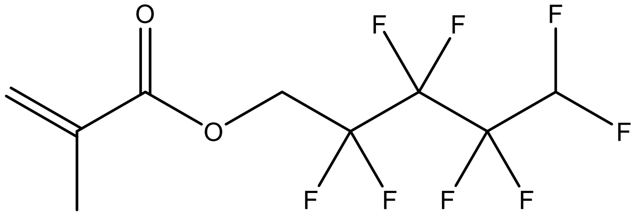
TMPTA	Trimethylolpropane triacrylate	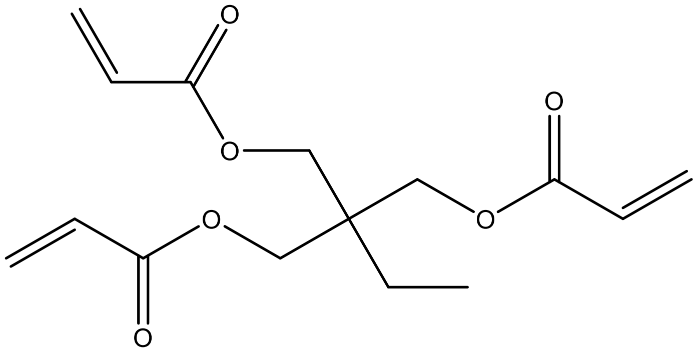
SMA	Stearyl methacrylate	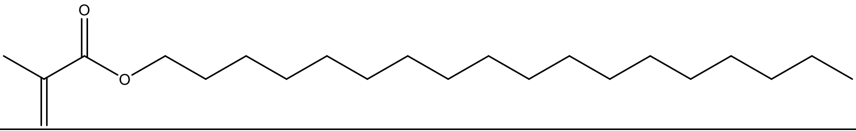

Supplement S2: MS^1 spectrum for an insulin digest

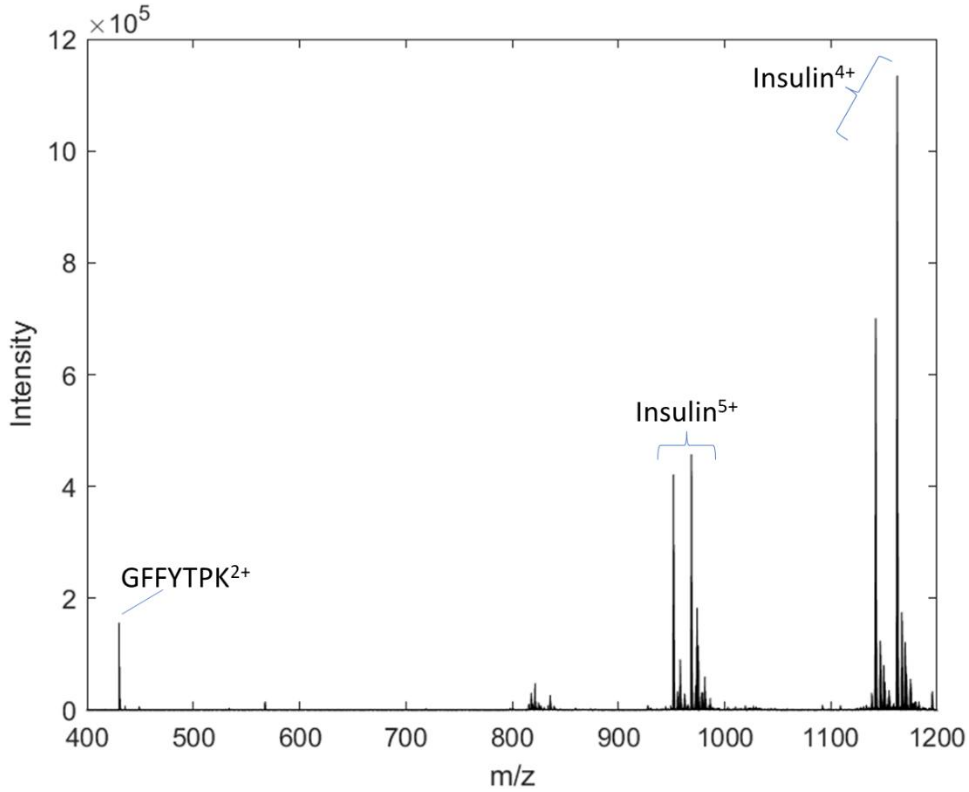

Supplementary Figure S2. LESA-MS spectrum of partially digested insulin acquired on a Q-Exactive plus Orbitrap mass spectrometer.

Supplement S3: Optimisation of digestion and LESA parameters for the analysis of peptides

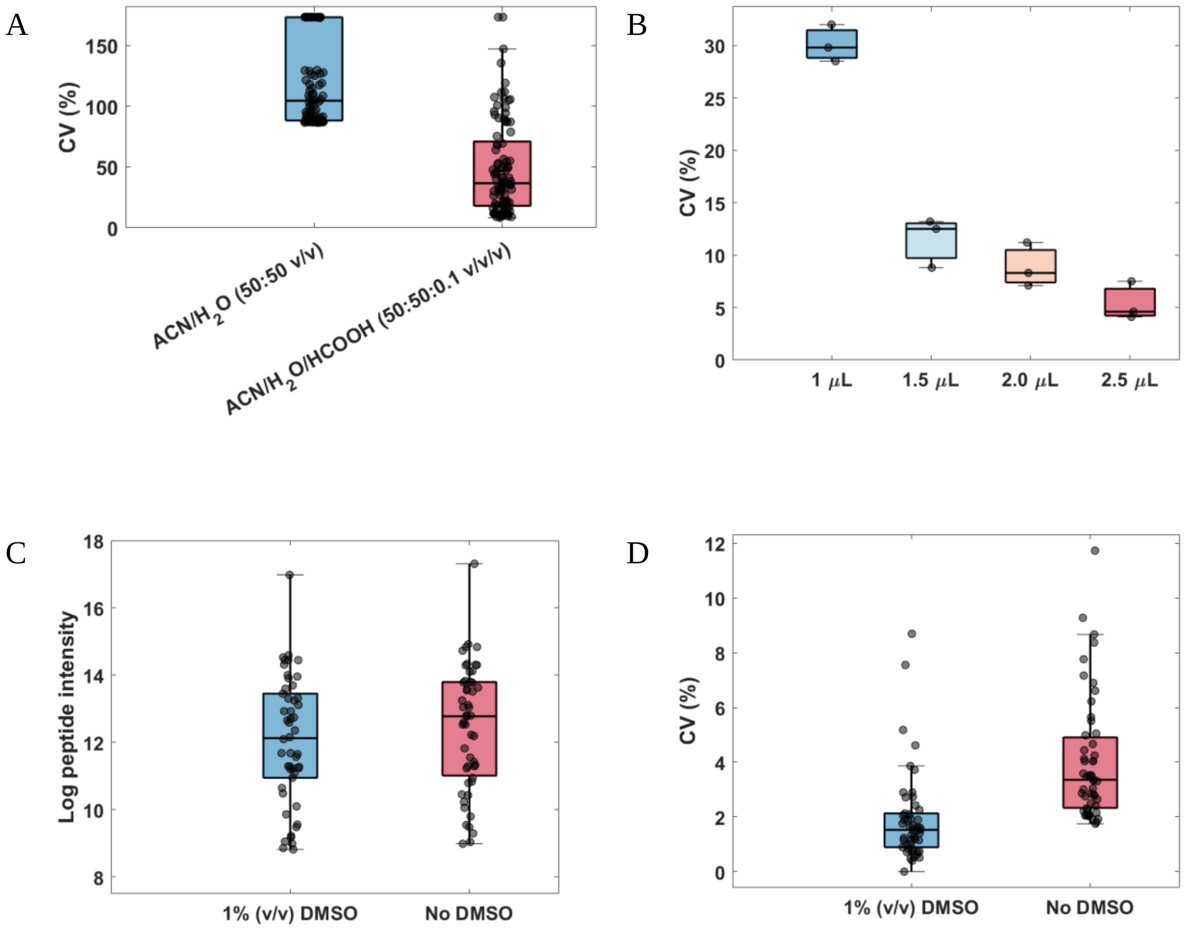

Supplementary Figure S3. Optimization of LESA-MS/MS parameters using BSA. (A) Assessment of formic acid as extraction solvent additive for repeatability of BSA peptide extraction/signal intensity (n = 3). (B) Selection of total solvent volume for peptide extraction (n = 3). (C) Testing DMSO as additive to improve extraction of hydrophobic peptides (n = 4). (D) Comparison of extraction repeatability using DMSO as solvent additive. Box and whiskers represent the median and quartiles of the data (n =4).

Supplement S4: Overview of significant Dragon descriptors

Supplementary Table S4: List of Dragon descriptors with description

Name	Description
Eig14_EA(dm)	Eigenvalue n. 14 from edge adjacency mat. weighted by dipole moment
DLS_07	modified drug-like score from Veber et al. (2 rules)
nR#CH/X	number of terminal C(sp)
H-054	H attached to C0(sp3) with 3X attached to next C
JGI4	mean topological charge index of order 4
MATS5m	Moran autocorrelation of lag 5 weighted by mass
JGI7	mean topological charge index of order 7
CATSD2D_00_DD	CATS2D Donor-Donor at lag 00
VE1sign_B(p)	coefficient sum of the last eigenvector from Burden matrix weighted by polarizability
Eig13_EA(dm)	eigenvalue n. 13 from edge adjacency mat. weighted by dipole moment
F06[O-O]	Frequency of C - O at topological distance 6
nArOR	number of ethers (aromatic)
P_VSA_e_5	P_VSA-like on Sanderson electronegativity, bin 5
nRNR2	number of tertiary amines (aliphatic)
Eig03_EA(dm)	eigenvalue n. 3 from edge adjacency mat.
GATS5i	Geary autocorrelation of lag 5 weighted by ionization potential
Eig11_AEA(dm)	eigenvalue n. 11 from augmented edge adjacency mat. weighted by dipole moment
B10[C-O]	Presence/absence of C - O at topological distance 10
P_VSA_LogP_6	P_VSA-like on LogP, bin 6
B03[O-Br]	Presence/absence of O - Br at topological distance 3
B05[N-O]	Presence/absence of N - O at topological distance 5
P_VSA_p3	P_VSA-like on polarizability, bin 3
